# Forage Carbohydrate Profiles and Endocrine Morphometric Interactions in Traditionally Managed Horses from Romania

**DOI:** 10.3390/life15111721

**Published:** 2025-11-06

**Authors:** Zsofia Daradics, Maria Popescu, Cornel Cătoi, Mircea Valerian Mircean, Adrian Macri, Oana Mîrza, Andrei Szakacs, Sorana Daina, Florinela Fetea, Mirela Alexandra Tripon, Alexandru Florin Lupșan, Denisa Bungărdean, Anamaria Călugăr, Florin Dumitru Bora, Cristian Mihăiță Crecan

**Affiliations:** 1 Department of Internal Medicine, Faculty of Veterinary Medicine, University of Agricultural Sciences and Veterinary Medicine (UASVM) Cluj-Napoca, Mănăştur Street 3-5, 400372 Cluj-Napoca, Romania; sofia.daradics@usamvcluj.ro (Z.D.); mircea.mircean@usamvcluj.ro (M.V.M.); 2 Equine Clinic, Faculty of Veterinary Medicine, University of Agricultural Sciences and Veterinary Medicine (UASVM) Cluj-Napoca, Mănăştur Street 3-5, 400372 Cluj-Napoca, Romania; maria.popescu@usamvcluj.ro; 3 Department of Pathology, Faculty of Veterinary Medicine, University of Agricultural Sciences and Veterinary Medicine (UASVM) Cluj-Napoca, Mănăştur Street 3-5, 400372 Cluj-Napoca, Romania; cornel.catoi@usamvcluj.ro; 4 Department of Animal Nutrition, Faculty of Veterinary Medicine, University of Agricultural Sciences and Veterinary Medicine (UASVM) Cluj-Napoca, Mănăştur Street 3-5, 400372 Cluj-Napoca, Romania; adrian.macri@usamvcluj.ro (A.M.); andrei.szakacs@usamvcluj.ro (A.S.); sorana.matei@usamvcluj.ro (S.D.); 5 Department of Bromatology, Hygiene, Nutrition, Faculty of Pharmacy, “Iuliu Haţieganu” University of Medicine and Pharmacy, 6 Louis Pasteur, 400349 Cluj-Napoca, Romania; oana.stanciu@umfcluj.ro; 6 Department of Food Science, Faculty of Food Science, University of Agricultural Sciences and Veterinary Medicine (UASVM) Cluj-Napoca, Mănăştur Street 3-5, 400372 Cluj-Napoca, Romania; florinela.fetea@usamvcluj.ro; 7 Department of Reproduction, Obstetrics and Veterinary Gynecology, Faculty of Veterinary Medicine, University of Agricultural Sciences and Veterinary Medicine (UASVM) Cluj-Napoca, Mănăştur Street 3-5, 400372 Cluj-Napoca, Romania; mirela.tripon@usamvcluj.ro; 8 Department of Anesthesiology and Surgery, University of Agricultural Sciences and Veterinary Medicine (UASVM) Cluj-Napoca, Mănăştur Street 3-5, 400372 Cluj-Napoca, Romania; alexandru-florin.lupsan@usamvcluj.ro (A.F.L.); cristian.crecan@usamvcluj.ro (C.M.C.); 9 Department of Pathophysiology, Faculty of Veterinary Medicine, University of Agricultural Sciences and Veterinary Medicine (UASVM) Cluj-Napoca, Mănăştur Street 3-5, 400372 Cluj-Napoca, Romania; denisa.bungardean@usamvcluj.ro; 10 Viticulture and Oenology Department, Advanced Horticultural Research Institute of Transylvania, Faculty of Horticulture and Business in Rural Development, University of Agricultural Sciences and Veterinary Medicine (UASVM) Cluj-Napoca, Mănăştur Street 3-5, 400372 Cluj-Napoca, Romania; anamaria.calugar@usamvcluj.ro; 11 Laboratory of Chromatography, Advanced Horticultural Research Institute of Transylvania, Faculty of Horticulture and Business for Rural Development, University of Agricultural Sciences and Veterinary Medicine (UASVM) Cluj-Napoca, Mănăştur Street 3-5, 400372 Cluj-Napoca, Romania

**Keywords:** equine metabolic health, forage composition, fermentable carbohydrates, insulin resistance, leptin, adiponectin, body condition scoring, neck crest adiposity, traditional horse husbandry, laminitis susceptibility

## Abstract

Horses maintained under traditional management systems and dependent on natural forages are often exposed to seasonal and compositional variations that can affect metabolic homeostasis. This study examined associations between forage nutrient composition and metabolic–morphometric indicators in horses from four agroecologically distinct regions of northwestern Romania. Eighty-eight horses managed under semi-extensive rural conditions underwent clinical examination, body condition scoring (BCS), cresty neck scoring (CNS), and fasting blood sampling. Forage samples (*n* = 34) from daily rations were analyzed for fermentable carbohydrate content, while serum insulin, leptin, and adiponectin were quantified using validated equine-specific ELISA assays. Forage composition varied substantially among regions, influencing both endocrine and morphometric outcomes. Horses consuming carbohydrate-rich forages exhibited higher insulin (0.95–219 μIU/mL) and leptin concentrations (925–28,190 pg/mL), accompanied by elevated BCS and CNS scores, whereas adiponectin levels tended to decrease with increasing carbohydrate content. These findings demonstrate that naturally occurring variation in forage quality can significantly influence metabolic regulation in horses managed under low-input, traditional systems. Integrating forage nutrient evaluation with clinical and endocrine assessments provides a practical framework for identifying animals at risk of metabolic dysfunction and guiding nutritional strategies to mitigate the incidence of laminitis and related disorders.

## 1. Introduction

Equine Metabolic Syndrome (EMS) is a clinical disorder characterized by insulin dysregulation (ID), regional adiposity, and an increased risk of endocrinopathic laminitis [1,2,3]. While laminitis is the main clinical manifestation, recent research has highlighted early metabolic disturbances—particularly insulin dysregulation—as central to the pathogenesis of EMS [4,5,6]. Furthermore, EMS shares several features with human Metabolic Syndrome (MetS) but exhibits specific traits in horses, including regional fat accumulation (neck, tail base, shoulders, and mammary region), intermittent hyperinsulinemia, insulin resistance, and associated complications such as hypertriglyceridemia, dyslipidemia, hypertension, and reproductive problems in mares [7,8]. Adipose tissue acts as an endocrine organ, releasing adipokines—such as leptin, adiponectin, and resistin—and pro-inflammatory cytokines (IL-1, IL-6, TNF-α), thereby contributing to low-grade chronic inflammation [7,9].

Insulin dysregulation represents the key metabolic abnormality of EMS, characterized by elevated basal insulin concentrations and exaggerated postprandial responses to oral or intravenous glycemic challenges [8,10,11,12]. Current evidence indicates that an excessive postprandial insulin surge—rather than insulin resistance alone—is the main driver of endocrinopathic laminitis [13,14,15,16,17]. Consequently, high-glycemic feeds rich in non-structural carbohydrates (NSC) are excluded from dietary management programs for EMS [11,18,19,20,21]. While the effects of starch and simple sugars are well documented, the metabolic impact of forage-derived carbohydrates—particularly fructans and water-soluble carbohydrates (WSC)—remains less clearly defined [20,22,23,24,25]. Fructans, abundant in cool-season grasses, are fermentable carbohydrates that can influence both insulin and adipokine responses [20,22,23,24,25]. Some horses may maintain normoglycemia through compensatory hyperinsulinemia, thereby masking metabolic dysfunction [20]. Moreover, early-life nutritional exposures may shape these regulatory mechanisms, suggesting a form of metabolic programming with long-term consequences [20].

Nutritional management is therefore critical for the prevention and control of EMS, as it directly influences insulin sensitivity and the risk of laminitis [26]. However, consistent associations between forage type, nutrient composition, and metabolic outcomes remain difficult to demonstrate under field conditions [20]. Some horses exhibit endocrine disturbances without apparent changes in body condition, underscoring the limited sensitivity of traditional scoring systems and the potential value of complementary tools such as ultrasonography or biomarkers including leptin and adiponectin [20,27]. Moreover, considerable interindividual variability observed under standardized feeding regimes suggests an additional influence of genetic and physiological factors [20,28].

Regional and environmental factors further modulate the risk of metabolic disorders in horses. In the four study areas of northwestern Romania—Maramureș, Bihor, Cluj, and Turda—horses are traditionally managed under low-input systems and rely mainly on semi-natural grasslands and hay. These regions, situated at altitudes between 200 and 600 m and characterized by a temperate-continental climate, include permanent meadows dominated by cool-season grasses such as *Festuca rubra* and *Agrostis capillaris* [29]. Such forages exhibit considerable variability in carbohydrate content, which may influence the risk of insulin dysregulation [29,30,31]. In addition, local forages and medicinal plants are rich in polyphenols and antioxidants that could modulate inflammatory and endocrine pathways [32,33,34]. Furthermore, the grasslands in these areas harbor diverse microbial flora that can affect forage fermentation, preservation, and nutrient composition, potentially influencing metabolic responses in grazing horses [35,36,37]. These ecological and management characteristics form the background for the present investigation.

Unlike Western Europe, where horses are primarily used for sport and leisure, Romanian horses remain essential for small-scale farming, forestry, and household transport. Managed under semi-traditional systems and relying almost entirely on pasture and hay, these animals are particularly vulnerable to dietary imbalances [29]. Under such conditions, evaluating the quality and carbohydrate composition of forage is crucial. The glycemic index (GI), originally developed for human nutrition, has been adapted for equine diets; however, nutrient composition—such as NSC and starch—alone does not fully predict glycemic or insulinemic responses [38,39]. Moreover, not all laminitic horses are obese or insulin resistant, emphasizing the multifactorial nature of the condition, which involves interactions among genetics, seasonality, diet, and neuroendocrine factors [40,41,42].

This study was designed to investigate, within a cross-sectional observational framework, the associations between forage carbohydrate composition—particularly fructans and water-soluble carbohydrates (WSC)—and both endocrine and morphometric indicators of EMS risk in crossbred draft-type horses from rural Romania. We specifically hypothesized that forages with higher fructan content would be associated with increased insulin and leptin but decreased adiponectin concentrations, and that cervical morphometry—assessed through cresty neck score and ultrasonographic subcutaneous fat thickness—would provide a more sensitive measure of regional adiposity than body condition scoring alone. The findings are exploratory and intended to provide a foundation for future longitudinal studies addressing environmental, nutritional, and management factors contributing to EMS [43,44,45,46].

## 2. Materials and Methods

All procedures were conducted between October and November 2019 under semi-controlled field conditions that reflected rural management practices. Clinical examinations, body condition score (BCS) and cresty neck score (CNS) assessments, fasting blood sampling, and forage collection from daily rations were performed. The study regions were selected for their high equine density and agroecological diversity. Sampling site GPS coordinates were recorded but are withheld for confidentiality. Non-invasive procedures (clinical examination, body condition and cresty neck scoring, morphometric measurements, cervical ultrasonography, and forage sampling) were exempt from formal ethical approval under national and institutional regulations in force at that time. Blood samples were collected by the official local veterinary practitioner as part of routine clinical and biochemical monitoring, performed with the owners’ consent and under legal veterinary supervision. These procedures were part of regular health assessments, involved no experimental intervention, pain, or distress, and were fully compliant with national veterinary legislation.

Procedures involving biological sampling (overnight fasting blood collection for endocrine biomarkers) required formal approval and were authorized by the Bioethics Committee of the University of Agricultural Sciences and Veterinary Medicine, Cluj-Napoca (Approval Code: 221/17 August 2020). Owner consent was obtained for all animals prior to participation. All procedures were conducted in accordance with the principles of animal welfare and the ARRIVE guidelines.

### 2.1. Animals and Clinical-Morphometric Evaluation

The study was conducted in four regions of northwestern Romania (Maramureș, Bihor, Cluj, and Turda), selected for their high equine density and agroecological diversity, which provided a representative picture of traditional horse management. Horses (*n* = 88, SGR crossbreds) were recruited from private households and semi-intensive units with seasonal pasture access and forage-based diets.

These SGR crossbreds represent a distinct local genetic resource established through structured breeding programs in northwestern Romania that systematically crossed native semi-heavy draft horses with lighter traction and Warmblood-type sport lines. The objective of this crossbreeding was to combine the robustness, endurance, and adaptability of indigenous draft horses with the improved conformation, versatility, and metabolic efficiency of lighter breeds. As a result, SGR crossbreds exhibit intermediate body size and musculoskeletal development, making them suitable for multipurpose use. They are widely employed in rural communities for agricultural traction, forestry, transport, and, to a lesser extent, recreational riding, reflecting both their economic importance and their suitability as a model population for metabolic and nutritional studies.

Between October and November 2019, all animals underwent clinical examination, body condition and cresty neck scoring, blood sampling, forage sampling, and cervical ultrasonography under consistent field conditions with minimal restraint to reduce stress. Forage samples from each horse’s daily ration were analyzed for botanical composition and proximate nutrients. The study regions have a temperate-continental climate and altitudes of 200–600 m, influencing forage composition and fructan content. Geospatial data were recorded to confirm environmental variation, with exact coordinates withheld for privacy.

The final sample included 88 horses, predominantly adults, with a small number of foals (<1 year old; *n* = 4) representing approximately 4.5% of the total cohort. The study population comprised 46 females and 42 males, aged between 1 and 18 years (mean ± SD = 8.6 ± 3.9 years), with an average body weight of 485 ± 62 kg. Detailed individual data on sex, age, and body weight are provided in Supplementary Table S6. These foals were fully weaned and maintained under the same management and feeding conditions as the adult horses and were subjected to the same clinical, endocrine, and morphometric assessments. Their inclusion was intentional, as it reflected real-world conditions of traditional horse management in rural Romania, and is consistent with previous field-based studies on equine metabolic health.

All horses underwent clinical examination prior to sampling, and animals with acute illness, systemic disease, or severe lameness were excluded. Baseline factors (age, sex ratio, and clinical condition) were comparable across regions, with no significant differences (*p* > 0.05; Supplementary Table S6). Regional characteristics of husbandry, feeding, and environment are summarized in Supplementary Table S1.

For each horse, metadata were systematically recorded, including geographic origin, breed classification (SGR crossbred or semi-heavy draft type), sex, age, feeding regimen, and laminitis status. Each animal was assigned a unique anonymized identifier to ensure traceability across all clinical, dietary, morphometric, and endocrine datasets. Nutritional data were initially collected through owner-reported questionnaires and subsequently validated through direct on-site inspection of feed components. Laminitis status was encoded as a binary variable (1 = positive clinical history or current signs; 0 = no evidence of laminitis). This variable was later used in the interpretation of endocrine and morphometric outcomes.

When clinical signs of laminitis were present, the severity of the condition was graded using the Obel system, which classifies laminitis into four grades (I–IV) based on locomotor patterns, stance, and digital pulse intensity.

Clinical, morphometric, and blood sampling procedures were performed in the morning before feeding by the same team using the same equipment, ensuring methodological consistency while reflecting real-world management. Regional differences in management and forage practices were as follows: Maramureș (*n* = 15)—horses were kept in semi-free traditional systems and used mainly for agricultural work, with on-site assessments minimizing transport stress. In Bihor (*n* = 18), management was also traditional, with forage sampled directly from daily rations to link diet and metabolic markers; horses were mainly used for labor and transport. In Cluj (*n* = 20), SGR crossbred horses—representing mixed local draft and light breeds—were used for both work and recreation, with evaluations conducted under partially standardized conditions. Specifically, horses were maintained under comparable feeding regimes (natural hay and concentrates offered at fixed times), semi-intensive housing (regular access to both shelter and pasture), and light work usage. In Turda (*n* = 35), the largest cohort reflected semi-intensive management with variable supplementation, including concentrates. Together, these regions provided environmental, nutritional, and metabolic data under real-world conditions, strengthening the relevance of findings for clinical screening and dietary recommendations in traditional horse systems.

Ultrasound assessment of cervical adiposity was performed immediately after blood collection, with the horses standing calmly and the neck held in a neutral position. NC and SFT were measured at three standardized, equidistant sites along the neck, defined as 25%, 50%, and 75% of the distance from the occiput to the highest point of the withers. These sites corresponded to the upper (NC-25%, SFT-25%), middle (NC-50%, SFT-50%), and lower (NC-75%, SFT-75%) regions, respectively (see Supplementary Figure S1).

At each site, NC was measured with a flexible tape, followed by ultrasound assessment of subcutaneous fat thickness (SFT) using a portable veterinary unit (Mindray Z5 Vet, 7.5 MHz linear transducer) (Mindray Bio-Medical Electronics Co., Ltd., Shenzhen, China). The probe was placed perpendicular to the ground over the fascial interface between the nuchal ligament and cervical muscles, identified by palpation and visual inspection.

At each site, three ultrasound images were taken, and subcutaneous fat thickness (SFT) was measured in millimeters using the device’s internal caliper. The mean value per site was used for analysis.

Body condition was scored independently by two trained evaluators using the 9-point Body Condition Score (BCS) scale, with the average score used to classify animals into BCS categories. Cresty Neck Score (CNS) was assessed using the 0–5 scale described by Carter et al. (2009) [47], also performed independently by two trained evaluators.

The intraobserver repeatability for ultrasound and morphometric measurements was assessed using intraclass correlation coefficients (ICC), all exceeding 0.96, confirming excellent reliability. Morphometric evaluation procedures followed the standardized protocol detailed in Appendix A.

### 2.2. Sampling and Analytical Evaluation of Forages and Nutrients

Inclusion rate was defined as the percentage of each forage or concentrate type in relation to the total ration provided to the horse. In this study, “natural hay” refers to hay harvested from permanent, traditionally managed meadows composed of diverse native grasses and herbs, without reseeding or intensive fertilization.

Following these definitions, forage samples (~2–3 kg) were collected during the same fieldwork visits, either directly from hay bales, loose forage piles, storage containers, or by hand-cutting representative portions of pasture (5–10 cm above ground level) in grazing areas accessible to the horses. For hay, subsamples (~500 g each) were taken from at least three different bales or storage points and pooled into a composite sample per horse group.

All samples were immediately placed in labeled resealable bags, assigned unique identifiers corresponding to the consuming horses, and stored under dry, shaded conditions until analysis. Botanical identification and chemical analyses were then performed to explore diet–metabolism associations, as described in the following sections.

In the laboratory, each sample was thoroughly homogenized and divided into subsets: one for botanical examination and another for nutritional analysis. For botanical identification, morphological structures (leaf shape, ligule type, stem architecture, inflorescence morphology) were examined macroscopically at the Animal Nutrition Laboratory, Faculty of Veterinary Medicine, Cluj-Napoca, Romania.

Observations were validated against authenticated herbarium specimens and regional botanical keys, with photographic documentation and inter-observer validation to ensure reproducibility. When species-level identification was not possible due to sample integrity or fragmentation, samples were classified into functional groups (grasses, legumes, forbs), a practical approach in heterogeneous traditional forage mixtures.

For nutritional evaluation, forage subsets were oven-dried at 60 °C, or air-dried at room temperature until a constant weight was achieved, then gently disaggregated and ground to a fine powder. Nutritional composition was assessed using classical wet chemistry methods, including spectrophotometric fructan assays, Soxhlet extraction for crude fat, and Fehling’s titration for reducing sugars (expressed as a percentage of dry matter, using glucose as the standard).

All analyses were carried out in duplicate, with quality control thresholds set at CV ≤ 10% (see Supplementary Table S3). Special emphasis was placed on identifying fructan-rich grasses and protein- or lipid-rich legumes, given their relevance to equine metabolism.

This combined botanical–chemical approach provided an ecologically relevant and analytically robust characterization of forage composition. These results supported the interpretation of dietary influences on endocrine and morphometric outcomes in the study horses.

Forage chemical composition was determined using classical methods adapted for equine nutrition (Supplementary Table S3). Air-dried samples were homogenized, finely ground, and sieved through a 1 mm mesh to ensure uniform particle size. Crude fat was quantified using Soxhlet extraction with petroleum ether, reducing sugars by Fehling’s titration, and fructans by hot water extraction (80 °C), enzymatic hydrolysis, and spectrophotometric quantification at 510 nm using calibration curves.

All assays were performed in duplicate, with blanks and internal standards for quality control. Nutrient profiles obtained from these analyses were used to explore correlations with endocrine and morphometric data. As fructan concentrations vary with environmental conditions and growth stage, the single forage measurement per region should be considered a snapshot of nutrient composition—a limitation that is further discussed in the Discussion section.

All reagents were of analytical grade, and forage nutrient analyses were performed using validated AOAC methods. Specifically, crude fat was quantified using Soxhlet extraction, reducing sugars using Fehling’s titration, and fructans using enzymatic hydrolysis followed by spectrophotometric quantification. Detailed information on reagents, assay kits, and calibration procedures is provided in Supplementary Table S3.

### 2.3. Blood Sample Collection, Storage, and Analysis

Biological sampling via jugular venipuncture (10 mL of blood) was performed between 7:00 and 9:00 a.m. after an overnight fast of 8–10 h. Blood sampling was carried out in the animals’ home environments, with calm restraint using non-invasive halters. During this period, forage was withheld, but water remained available ad libitum. This procedure was feasible under semi-extensive management, as animals were routinely stabled overnight.

Approximately 10 mL of blood was drawn into serum separator tubes (Vacutainer^®^, BD Diagnostics, Franklin Lakes, NJ, USA) containing a clot activator and polymer gel. Tubes were gently inverted 5–8 times and left upright at room temperature (20–25 °C) for 30–60 min to allow clotting. Samples were then centrifuged at 1500–2000× *g* for 10–15 min. Serum was carefully separated using sterile pipette tips (Eppendorf AG, Hamburg, Germany) and transferred into labeled cryogenic vials (Eppendorf AG, Hamburg, Germany) (1.5–2.0 mL).

Following serum separation, aliquots were processed in a fully equipped mobile veterinary clinic adapted for field conditions, including a centrifuge (Eppendorf AG, Hamburg, Germany), portable freezers (−20 °C and −80 °C), (Thermo Fisher Scientific, Waltham, MA, USA) and Dewar tanks (MVE Biological Solutions, New Prague, MN, USA) with liquid nitrogen. Samples were immediately placed either in portable freezers or in liquid nitrogen, depending on the expected time until shipment, and were maintained under these controlled conditions for a maximum of 8 h. Subsequently, all aliquots were transported on dry ice directly to the University laboratory, where they were uniformly stored at −80 °C until analysis. This procedure ensured strict maintenance of the cold chain and methodological consistency across all sampling sites.

Before ELISA analysis, serum samples were thawed once at 4 °C, mixed gently, and used immediately to preserve biomarker stability and ensure consistent sample quality under field conditions.

Although the original determination of the glycemic index (GI) in horses, as described by Rodiek et al. (2007) [38], involves postprandial glucose monitoring over several hours, such procedures were not feasible under field conditions. Therefore, this study focused on proxy indicators of glycemic response and energy metabolism—namely fasting insulin levels, morphometric adiposity markers, and serum concentrations of leptin and adiponectin—as practical alternatives for assessing metabolic risk [48].

Metabolic biomarkers (insulin, leptin, and adiponectin) were quantified using equine-specific ELISA kits (MyBioSource, San Diego, CA, USA; Cat. No. MBS701443 for insulin, MBS701441 for leptin, and MBS701445 for adiponectin), following the manufacturer’s protocols to ensure analytical accuracy. Optical densities were measured at 450 nm using a microplate reader (BioTek ELx808, Winooski, VT, USA). Supplementary Table S2 provides full technical details, including detection limits, assay ranges, variability coefficients, recovery rates, and dilution factors. Frozen serum aliquots were thawed overnight at 4 °C, equilibrated to room temperature, and gently mixed before analysis. To prevent degradation, aliquots were used only once. All samples and standards were run in duplicate, and intra- and inter-assay variability were monitored (<10% and <15%, respectively). Assays exceeding these thresholds were repeated, and duplicate measurements differing by more than 15% were reanalyzed whenever possible.

### 2.4. Statistical Analysis

A priori power analysis was conducted using G*Power 3.1 (Heinrich Heine University, Düsseldorf, Germany) to determine the minimum required sample size. Parameters were set for a two-tailed correlation test, with α = 0.05, power (1–β) = 0.80, and an expected medium effect size (ρ ≈ 0.32), based on previous studies in equine metabolic research. This power analysis was specifically designed for correlation tests between forage nutrient composition and endocrine biomarkers (insulin, leptin, and adiponectin).

The morphometric ultrasound subset (*n* = 34) included only those horses with complete imaging datasets and was analyzed descriptively and exploratorily rather than as a separately powered sample. This smaller subset reflects the logistical feasibility of field-based imaging procedures and, while it does not affect the main analytical framework, represents a limitation in terms of statistical power for morphometric comparisons.

All statistical analyses were performed using IBM SPSS Statistics version 29 (IBM Corp., Armonk, NY, USA) and GraphPad Prism version 10 (GraphPad Software, La Jolla, CA, USA). Prior to analysis, the distributions of serum biomarkers (insulin, leptin, and adiponectin) were assessed using quantile–quantile (Q–Q) plots and the Shapiro–Wilk test to evaluate normality. Given the right-skewed nature of the raw concentration data, log_10_ transformation was applied to all biomarker values to normalize distributions and satisfy assumptions for parametric testing. Descriptive statistics (mean, standard deviation, minimum, and maximum) were computed for all dietary and morphometric variables, while categorical variables were expressed as counts and percentages.

Associations between forage nutrient composition—particularly fructan, fat, and carbohydrate content—and both morphometric and metabolic outcomes were evaluated using Pearson’s correlation coefficient (r) or Spearman’s rank correlation coefficient (ρ), as appropriate. Correlation strength was interpreted as negligible (<0.30), low (0.30–0.49), moderate (0.50–0.69), or strong (≥0.70). Correlation matrices were visualized as heatmaps to facilitate interpretation. For statistical purposes, laminitis was coded as a binary variable (present/absent).

Comparisons of continuous outcomes (e.g., SFT) across categorical predictors (e.g., BCS category, forage type) were performed using one-way analysis of variance (ANOVA) when assumptions for parametric testing were met. SFT values from the three cervical regions were compared across BCS categories to assess patterns of regional adiposity. Homogeneity of variances was evaluated using Levene’s test. When this assumption was met (*p* ≥ 0.05), ANOVA was followed by Tukey’s honestly significant difference (HSD) post hoc test for pairwise comparisons. When violated (*p* < 0.05), Welch’s ANOVA was applied instead, followed by Games–Howell post hoc testing. Effect sizes for ANOVA were expressed as partial eta squared (η^2^p).

Potential confounding variables, including breed, sex, location, feeding practices, and physiological status (e.g., gestation), were recorded but not formally included in the statistical models due to sample size limitations. Information on regional climatic conditions considered during data interpretation is provided in Appendix B. Additionally, no standardized diagnostic tests for EMS were performed; therefore, comparisons between horses with and without EMS were not statistically feasible. Nevertheless, we sought to verify whether the studied population was demographically and regionally homogeneous before performing correlation analyses.

Potential confounding variables (age, sex, breed, and region) were formally assessed to verify population homogeneity prior to the main analyses. Depending on distributional assumptions, parametric (one-way ANOVA) or non-parametric (Kruskal–Wallis, Mann–Whitney) procedures were applied, while associations with continuous covariates such as age were evaluated using Spearman’s rank correlation. Data normality and homoscedasticity were verified using the Shapiro–Wilk and Levene tests, respectively. None of the tested demographic or regional factors exhibited statistically significant effects on endocrine or morphometric variables (all *p* > 0.05; Supplementary Table S8), confirming the structural homogeneity of the study population. Consequently, pairwise correlations were retained as an appropriate exploratory approach for identifying field-relevant associations under semi-controlled conditions.

A significance threshold of *p* < 0.05 (two-tailed) was adopted for all statistical tests. Graphical representations—including boxplots, heatmaps, and scatterplots—were generated using both SPSS and GraphPad Prism to visually support the interpretation of key findings. Finally, in addition to univariate analyses, multivariate correlation analyses were performed to evaluate associations among forage nutrient composition, morphometric variables (BCS, CNS, SFT), and endocrine biomarkers (insulin, leptin, adiponectin).

## 3. Results

The a priori power analysis (two-tailed correlation, α = 0.05, 1–β = 0.80, expected effect size ρ ≈ 0.32) indicated a minimum required sample size of 76 horses. The final cohort included 88 individuals, thus exceeding this threshold and ensuring sufficient statistical power for the conducted analyses.

The study cohort comprised 88 horses (84 adults and 4 weaned foals) originating from four regions of northwestern Romania (Maramureș, Bihor, Cluj, and Turda). The population consisted of SGR crossbreds (*n* = 33) and semi-heavy draft horses (*n* = 55), reflecting the typical breed composition of working equines in traditional management systems. Females predominated (77.3%), while the remainder were males or geldings. The mean age of the horses was 6.3 ± 3.9 years (range: 0.5–15 years), with comparable distributions across the four study areas (*p* > 0.05). No significant differences were observed between regions in age, sex ratio, or general clinical condition. Detailed individual data, including horse ID, farm location, breed, sex, age, feeding management, and laminitis status, are provided in Supplementary Table S6.

Additional inferential analyses were conducted to verify the influence of demographic and regional covariates on endocrine and morphometric variables. Statistical testing revealed no significant effects of age, sex, breed, or region on fasting insulin, leptin, adiponectin, body condition score (BCS), or cresty neck score (CNS) (all *p* > 0.05; Supplementary Table S8). These findings confirm the statistical homogeneity of the study population and indicate that the observed pairwise correlations reflect genuine field-level associations rather than confounded effects.

### 3.1. Botanical Composition of Forage and Concentrate Components in Equine Diets

The feed composition of the study horses (*n* = 88) is presented in Table 1. Alfalfa hay and natural hay were the most frequently used forages, with mean inclusion rates (percentage contribution of each forage or concentrate type to the total ration) of 78.6% (SD = 24.45) and 85.27% (SD = 14.42), respectively. Mixed hay (alfalfa, clover, grass) was provided to 7 horses, exclusively at 100% inclusion. Grass-based forages were offered to 23 horses, with a mean inclusion of 20.78% (SD = 11.12). Other forage components included medicinal plants (mean = 5.26%, SD = 3.53), chicory (mean = 6.33%, SD = 2.29), fresh alfalfa (mean = 7.08%, SD = 10.37), legumes (mean = 14.45%, SD = 1.51), corn leaves (mean = 17.5%, SD = 6.12), and mixed ryegrass (mean = 47.5%, SD = 35.0).

Among concentrates, oats were administered to 13 horses with near-uniform inclusion (mean = 99.54%, SD = 1.13). Ground corn and barley showed greater variability, with mean inclusion rates of 26.2% (SD = 15.05) and 67.0% (SD = 9.49), respectively. Wheat bran was used in smaller amounts (mean = 13.6%, SD = 3.13). A distinct concentrate category, mixed mash (corn, barley, oats), was identified in 24 horses, consistently at 100% inclusion.

The term mixed hay refers to a forage mixture composed of alfalfa, clover, and grass, administered as the sole forage source in the diets of some horses. The term mixed mash denotes a concentrate blend containing ground corn, barley, and oats in variable proportions, prepared and offered as a single feed item.

**Figure 1:**
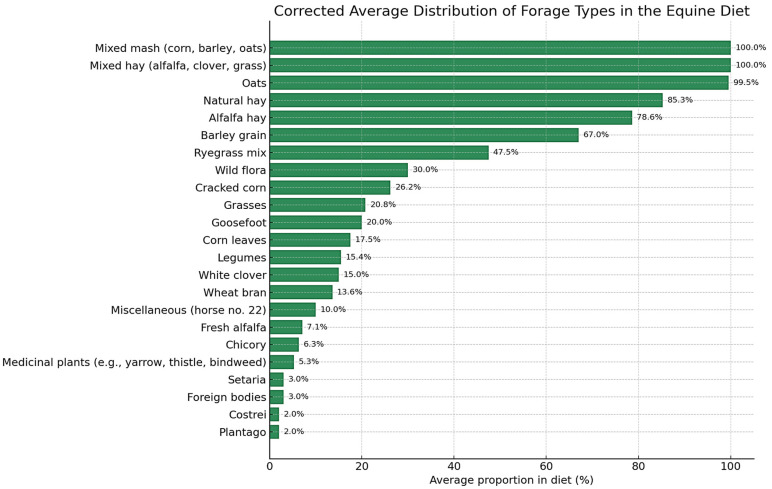
Distribution of Average Inclusion Rates for Forage and Concentrate Types in Equine Diets. Note: Figure 1 represents average dietary inclusion values based on feed type consumption in 88 horses. Only feed types with *n* ≥ 5 are included. Values are presented as mean ± SD (*n* = 88 horses).

Figure 1 illustrates the average inclusion rates of the main forage and concentrate types in the diets of the study horses. Alfalfa hay, natural hay, and mixed mash (corn, barley, oats) were the most frequently used dietary components, with some horses receiving them exclusively. Other items such as chicory, legumes, and medicinal plants were used more selectively and at lower inclusion rates, contributing to dietary variability across individuals.

This heterogeneity in feed composition reflects differences in regional management practices and may influence metabolic outcomes. Detailed botanical identification of forages, including classification by fructan accumulation potential, is provided in Supplementary Table S4. Forage components consumed by fewer than five horses were excluded from the main analyses but are summarized in Supplementary Table S5.

### 3.2. Serum Endocrine Biomarkers

**Figure 2:**
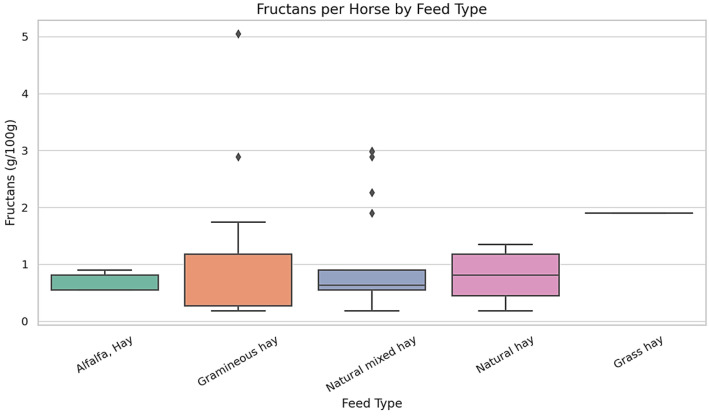
Fructan Content by Feed Type in Draft Horses (g/100 g DM). Note: Boxplots represent the distribution of fructan concentrations (g/100 g of dry matter) in different forage categories consumed by 88 horses. Only feed types with *n* ≥ 5 are included. Boxes indicate interquartile range (IQR), the horizontal line within each box shows the median, and whiskers represent the 5th–95th percentiles. Outliers are plotted as individual dots. Statistical differences among feed types were assessed using one-way ANOVA followed by Tukey’s post hoc test (*p* < 0.05).

Figure 2 presents the distribution of fructan concentrations (g/100 g dry matter) across five commonly used forage types in the diets of draft horses. The data demonstrate substantial variability in fructan content between forage types. Gramineous hay and natural mixed hay showed the widest interquartile ranges, along with several statistical outliers, suggesting inconsistency in fructan levels among different samples. In contrast, alfalfa hay exhibited a narrower range of values, with lower variability across samples. Ryegrass-based hays and pasture grasses also showed moderate variability, with fructan values falling within intermediate ranges. Overall, these findings reveal that fructan concentrations vary not only across forage types but also within the same forage category.

For each animal, dietary intake was assessed, with particular attention to the types and proportions of forage consumed. Fructan content in the ingested forages ranged from X to Y g per 100 g of dry matter, with a mean value of Z g/100 g DM. These values were recorded individually and aggregated to allow comparative evaluation across dietary patterns. Forage types varied in fructan content, contributing to the observed diversity in dietary carbohydrate exposure within the population.

**Figure 3:**
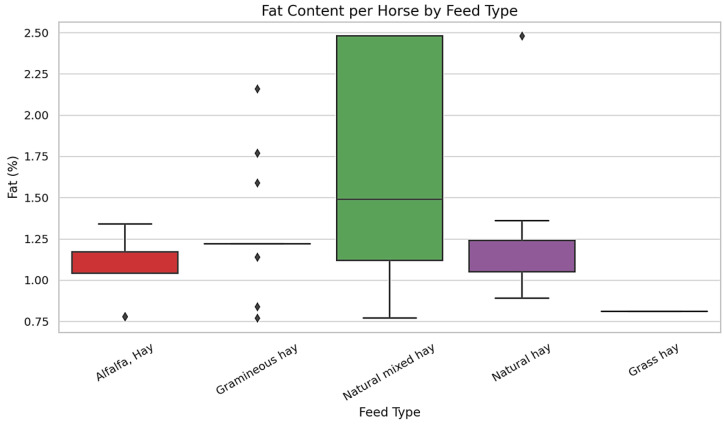
Fat Composition of Equine Forages by Feed Type. Note: Black diamond markers indicate outliers; none were removed from the dataset. Note: Boxplots illustrate the distribution of fat content (%) across different forage types consumed by 88 horses. Only feed types with *n* ≥ 5 are included. Boxes represent the interquartile range (IQR), the horizontal line indicates the median, and whiskers extend to the 5th–95th percentiles. Outliers are shown as individual points. Statistical differences between feed types were evaluated using one-way ANOVA followed by Tukey’s post hoc test (*p* < 0.05).

The horses in the study consumed various hay types, including Alfalfa Hay, Gramineous Hay, Natural Hay, Natural Mixed Hay, and Grass Hay. Alfalfa hay generally exhibited lower and more consistent fructan concentrations (mean = 0.55 g/100 g DM); however, its range (0.18–1.78 g/100 g DM) partially overlapped with that of Natural Hay (0.18–1.35 g/100 g DM), indicating that differences between the two forage types were not absolute. Gramineous Hay showed the widest range of fructan content (0.18 to 5.05 g/100 g), with the highest value recorded in a Light Draft Horse. Natural Mixed Hay also exhibited a broad concentration range (0.18 to 2.99 g/100 g), suggesting notable variation among samples. Natural Hay exhibited moderate and relatively stable fructan concentrations, ranging from 0.18 to 1.35 g/100 g. Grass Hay was represented by a single sample with a fructan value of 1.90 g/100 g. Fat concentrations (%) were measured across five commonly administered forage types. Natural Mixed Hay exhibited the widest range of fat content, while other forages showed narrower distributions. The detailed values for each forage category are presented in Figure 3.

Among the five forage types analyzed, Alfalfa Hay exhibited the most consistent and stable fat content, with concentrations tightly clustered between 1.13% and 1.17% of dry matter, and a single outlier at 1.34%. Although Grass Hay showed similar lipid consistency, its limited sample size precludes broader generalization. These results highlight the potential influence of forage type on dietary fat intake—a factor relevant to energy balance, thermoregulation, and metabolic stability. Despite comprising a minor fraction of total forage, crude fat contributes to the caloric density of equine diets and may be particularly important for horses with elevated energy demands or metabolic disorders such as Equine Metabolic Syndrome [49]. The observed consistency in Alfalfa Hay supports its use as a reliable nutritional component in controlled or therapeutic feeding programs.

In contrast, Natural Mixed Hay exhibited considerable variability in fat content, ranging from 0.77% to 2.48% of dry matter. This wide range suggests compositional heterogeneity. These results indicate that fat content differs substantially across forage types and batches, potentially reflecting underlying variation in botanical composition, forage maturity, or environmental conditions at harvest [50].

Natural Hay also showed considerable variability in fat content, with measured values ranging from 0.89% to 2.48% of dry matter. The highest fat value observed in this forage type equaled that of Natural Mixed Hay. Grass Hay, represented by a single sample (0.81%), fell at the lower end of the fat concentration range. Due to the limited sample size, no generalizations can be made for this forage category. Detailed distribution patterns are provided in Supplementary Table S7.

**Figure 4:**
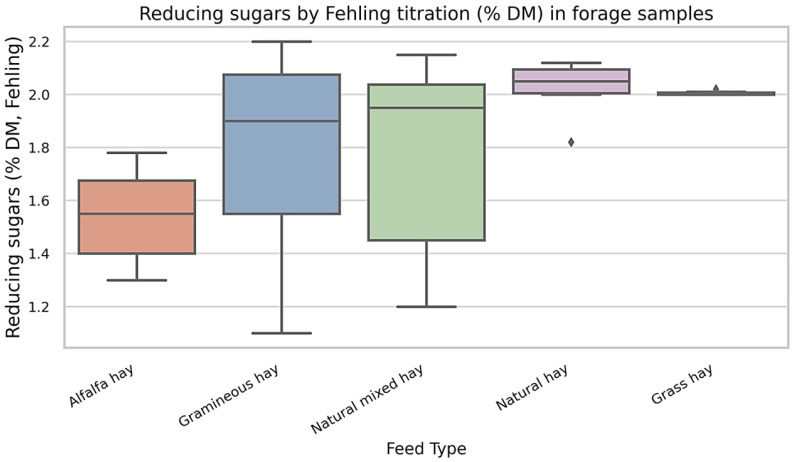
Reducing sugars by Fehling titration (% DM) in forage samples. Values represent only reducing sugars (e.g., glucose, fructose) determined by Fehling titration, not total carbohydrate or NSC content. Note: Boxplots show the distribution of reducing sugar concentrations (% of dry matter) in different forage types consumed by 88 horses. Only feed types with *n* ≥ 5 are included. Boxes represent the interquartile range (IQR), horizontal lines indicate median values, and whiskers correspond to the 5th–95th percentiles. Outliers are plotted as individual points. Statistical comparisons were conducted using one-way ANOVA followed by Tukey’s post hoc test (*p* < 0.05).

The distribution of reducing sugar content (% of dry matter, determined by Fehling titration) across five forage types is shown in Figure 4. Gramineous Hay and Natural Mixed Hay exhibited the widest ranges and highest variability in reducing sugar concentration, with multiple outliers and broad interquartile intervals. In contrast, Alfalfa Hay demonstrated consistently lower reducing sugar values with minimal dispersion among samples. These data indicate substantial differences in the readily fermentable carbohydrate fraction across forage types commonly fed to draft horses.

The analysis of total carbohydrate content (% of dry matter) in 88 forage samples administered to draft-type horses revealed inter-forage variability. Gramineous Hay and Natural Mixed Hay showed the widest range of carbohydrate concentrations, from 1.13% to 2.13%. In several cases, values were near the upper end of the observed spectrum. Natural Hay exhibited a narrower range (1.82–2.13%), while Grass Hay, represented by a single sample, showed a value of 2.01%. Fructan-rich diets were significantly associated with higher BCS, CNS, and SFT values (ANOVA, *p* < 0.05). Body condition scores (BCS) ranged from 4 to 9, with a median of 7 (Supplementary Table S7).

### 3.3. Serum Biomarker Distributions and Statistical Associations

**Figure 5:**
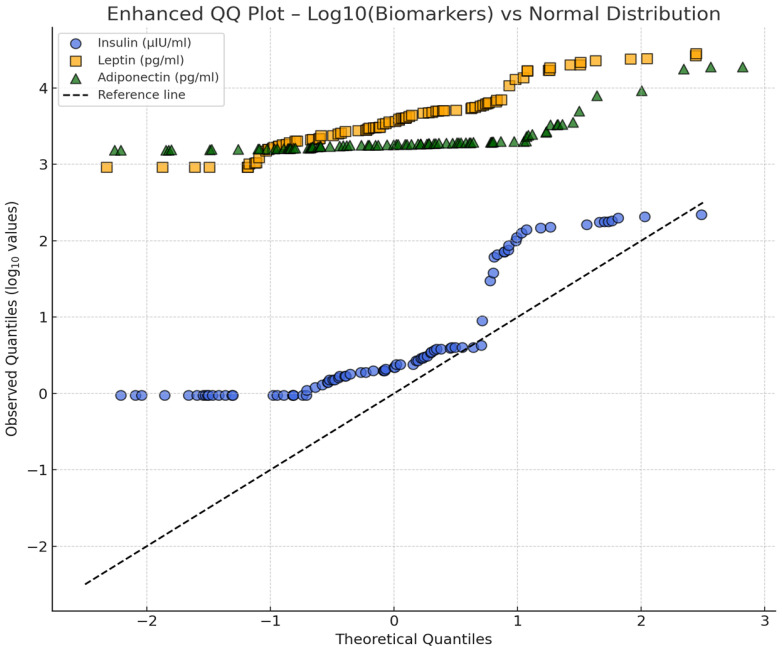
Quantile–Quantile plots of serum biomarkers (insulin, leptin, and adiponectin) in the study horses. Note: Q–Q plots illustrate the distributions of serum insulin, leptin, and adiponectin after log_10_ transformation. Each biomarker is compared visually with the expected normal distribution, showing deviations and variability across the study population. Note: The figure illustrates quantile–quantile (QQ) plots for log_10_-transformed values of Insulin (μIU/mL), Leptin (pg/mL), and Adiponectin (pg/mL) obtained from 88 horses. Each biomarker is compared to a theoretical normal distribution; deviations from the reference line indicate departures from normality. The log-transformation improved the distribution symmetry, supporting the use of parametric tests (ANOVA and correlation analysis) for further comparisons. Reference line = expected quantiles under normality.

Laminitis status was considered as a stratification factor in the interpretation of endocrine and morphometric outcomes (Figure 5).

Serum insulin concentrations (*n* = 88) ranged from 0.95 μIU/mL to 219 μIU/mL, with most horses exhibiting fasting values below 5 μIU/mL and a small subset showing markedly elevated levels (>100 μIU/mL). Serum leptin ranged from 925 pg/mL to 28,190 pg/mL, with the majority of values below 10,000 pg/mL but a subgroup exceeding 20,000 pg/mL. Adiponectin concentrations showed a bimodal pattern, with many horses clustering near the lower detection limit (1525 pg/mL), while others presented substantially higher values up to 18,860 pg/mL.

Across the cohort, higher fructan concentrations in forage were positively correlated with insulin (ρ = 0.45, *p* = 0.01) and leptin (ρ = 0.52, *p* = 0.004), and negatively correlated with adiponectin (ρ = −0.39, *p* = 0.03).

To further explore potential age-related differences, a stratified analysis was performed by age group (<5 years, ≥5 years). No significant differences were observed in the distribution of endocrine biomarkers (insulin, leptin, adiponectin) or morphometric parameters (BCS, CNS, SFT) between the two age categories (*p* > 0.05). Detailed values are provided in Supplementary Table S7.

### 3.4. Multivariate Assessment of Cervical Morphometry and Its Relationship with Body Condition in Crossbred Horses

This section reports the results of multivariate correlation analyses, integrating dietary, morphometric, and endocrine variables.

**Figure 6:**
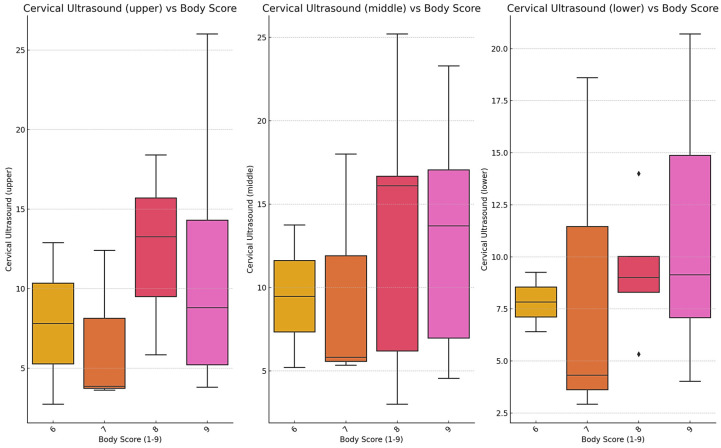
Boxplot Representation of Cervical Ultrasound Measurements (Upper, Middle, Lower) According to Body Condition Score (1–9). Note: Boxplots represent the distribution of ultrasound-measured fat thickness (mm) at three standardized neck positions (upper, middle, lower) according to body condition score (1–9 scale). Data were obtained from 34 horses with complete morphometric and ultrasound records. Boxes indicate the interquartile range (IQR), horizontal lines denote medians, and whiskers represent the 5th–95th percentiles. Outliers are shown as individual points. Statistical differences among BCS categories were tested using one-way ANOVA followed by Tukey’s post hoc comparison (*p* < 0.05). Box colors correspond to individual BCS categories (yellow = BCS 6, orange = BCS 7, red = BCS 8, pink = BCS 9) to facilitate visual comparison among condition groups.

Cervical morphometry was evaluated in 34 SGR crossbred horses with complete datasets for BCS, linear neck scores, and ultrasound-derived subcutaneous fat thickness (SFT). As shown in Figure 6, ultrasound measurements of the upper, middle, and lower cervical regions increased progressively with BCS. This trend was most evident in the middle and lower neck, where horses with BCS ≥ 8 consistently displayed higher fat thickness compared to those with moderate condition scores. Variability within BCS groups was also observed, reflecting interindividual differences in fat distribution.

**Figure 7:**
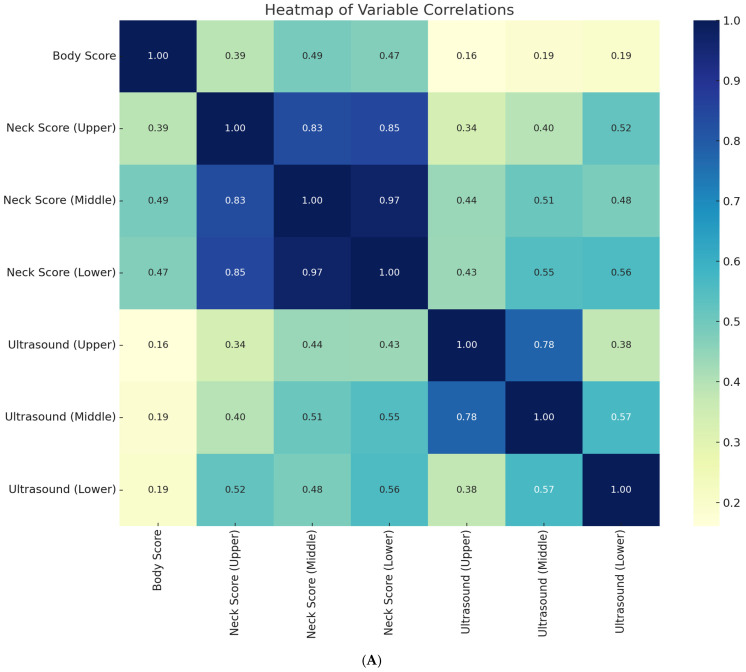

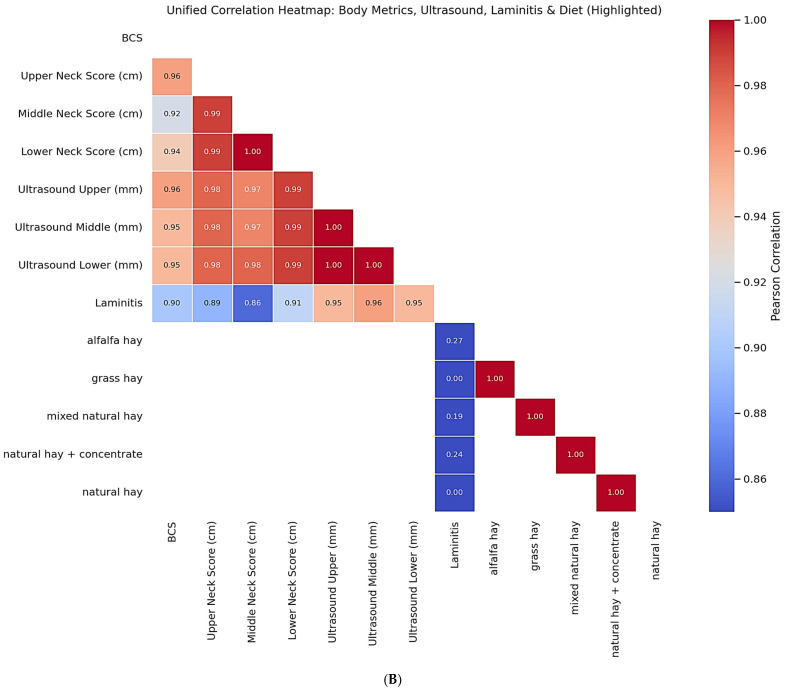
(**A**) Heatmap of Pearson Correlations Between Body Score, Neck Scores, and Cervical Ultrasound Measurements. Note: The heatmap displays Pearson correlation coefficients (r) between body condition score (BCS), neck scores (upper, middle, lower), and ultrasound-measured subcutaneous fat thickness at corresponding neck levels. The dataset includes 34 horses with complete morphometric and ultrasound measurements. Color intensity represents the strength of the correlation (blue = positive; yellow = weak). All correlations shown are statistically significant (*p* < 0.05). The strong positive relationships between neck scores and ultrasound values confirm the internal consistency and reliability of morphometric indicators for estimating regional adiposity. (**B**) Pearson correlation heatmap illustrating relationships among feeding regimen, laminitis incidence, body condition score (BCS), neck morphometry, and cervical ultrasound-derived adiposity metrics. Note: The heatmap presents Pearson correlation coefficients (r) among morphometric parameters (BCS and neck scores), ultrasound fat thickness (upper, middle, lower), laminitis occurrence, and predominant diet types. The analysis was performed on 88 horses with complete records. Color intensity indicates the direction and magnitude of correlations (red = strong positive; blue = negative). All values shown are statistically significant (*p* < 0.05). Strong positive correlations (r ≥ 0.90) between morphometric and ultrasound variables confirm consistency across body fat assessment methods, while the negative association between laminitis and certain diet types highlights the nutritional modulation of metabolic risk.

Correlation analysis confirmed strong intercorrelations among morphometric variables. Upper, middle, and lower cervical cresty neck scores (CNS) were strongly correlated (r = 0.83–0.97, *p* < 0.001), while ultrasound-derived subcutaneous fat thickness (SFT) values across cervical regions also demonstrated high concordance (upper–middle: r = 0.78, *p* < 0.001; middle–lower: r = 0.81, *p* < 0.001). Moderate associations were found between cresty neck scores and SFT, particularly in the lower cervical region (r = 0.56, *p* < 0.001). By contrast, body condition score (BCS) showed only weak correlations with ultrasound measures (r = 0.16–0.19, *p* > 0.05) and modest associations with cresty neck scores (r = 0.39–0.49, *p* < 0.05), indicating that traditional body condition scoring underestimates regional cervical adiposity (Figure 7A).

It should be acknowledged that breed-related genetic factors may influence neck morphometry and patterns of regional adiposity. In this study, all horses were local draft-type crosses managed under similar traditional systems, which likely reduced—but did not completely eliminate—breed-related variability. Future studies comparing different breeds under standardized conditions are warranted to clarify the role of genetic background in cervical fat deposition. Dietary patterns were reflected in morphometric outcomes. Horses receiving natural hay supplemented with concentrates exhibited the highest cervical SFT values, with consistent increases across all anatomical regions. In contrast, horses maintained on forage-only diets—particularly alfalfa hay or mixed natural hay—showed lower and more homogeneous measurements, rarely exceeding the mid-range of the distribution. These findings suggest that energy-dense feeding regimens contribute substantially to cervical fat accumulation.

The integrated analysis presented in Figure 7B highlights the relationships between diet type, laminitis status, morphometric indices, and ultrasound measures. Neck scores and ultrasound-derived values were nearly perfectly correlated (r = 0.95–1.00, *p* < 0.001), supporting their interchangeability as indicators of cervical adiposity. Laminitis status demonstrated strong positive associations with both morphometric (r = 0.86–0.91, *p* < 0.001) and ultrasound-derived adiposity measures (r = 0.95–0.96, *p* < 0.001), confirming the clinical link between localized fat deposition and metabolic risk. In addition, diet type correlated with laminitis: alfalfa hay (r = 0.27, *p* = 0.041), mixed hay (r = 0.19, *p* = 0.12), and hay plus concentrates (r = 0.24, *p* = 0.065) showed positive associations, whereas grass hay and natural hay alone did not.

The integrated analysis presented in Figure 7B showed strong correlations between neck scores and ultrasound-derived values (r = 0.95–1.00, *p* < 0.001). Laminitis status was positively associated with both morphometric measures (r = 0.86–0.91, *p* < 0.001) and ultrasound-derived adiposity measures (r = 0.95–0.96, *p* < 0.001). Regarding dietary influences, alfalfa hay (r = 0.27, *p* = 0.041), mixed natural hay (r = 0.19, *p* = 0.12), and hay plus concentrates (r = 0.24, *p* = 0.065) showed positive associations with laminitis, whereas grass hay and natural hay alone displayed no association (r ≈ 0.001).

Although the two correlation heatmaps display overlapping information, they represent different analytical levels the first focusing on internal morphometric consistency, and the second integrating dietary and metabolic variables.

### 3.5. Associations Between Endocrine Biomarkers and Morphometric Indicators

**Figure 8:**
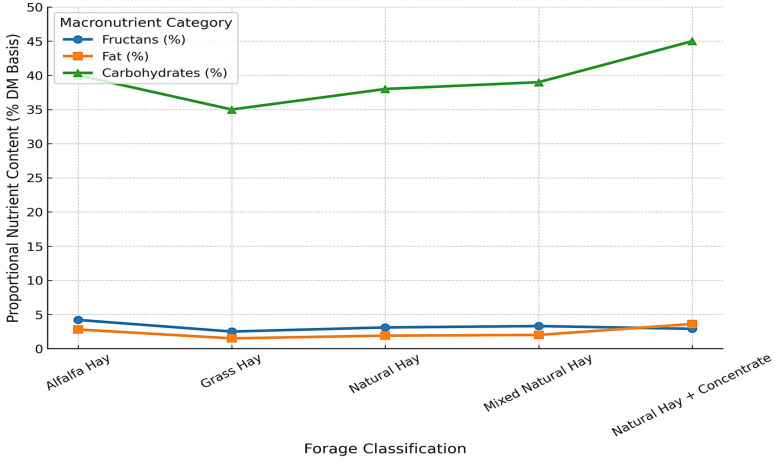
Nutritional Composition of Common Forage Types Used in Equine Feeding Programs. Note: The figure illustrates the proportional nutrient content (% dry matter basis) of different forage categories consumed by 88 horses. Data are expressed as mean values for fructans, fat, and carbohydrates in each feed type. Error bars represent standard deviations. Differences among forage types were evaluated using one-way ANOVA followed by Tukey’s post hoc test (*p* < 0.05). The higher carbohydrate content observed in “natural hay + concentrate” reflects increased energy density, while fructan and fat levels remained comparatively low and stable across feed categories.

Figure 8 illustrates the variability in macronutrient composition across common forage types. Diets based on natural hay plus concentrate showed higher carbohydrate and fat contents compared to single-source forages such as alfalfa hay and grass hay. Detailed descriptive values are presented in Table 2.

**Figure 9:**
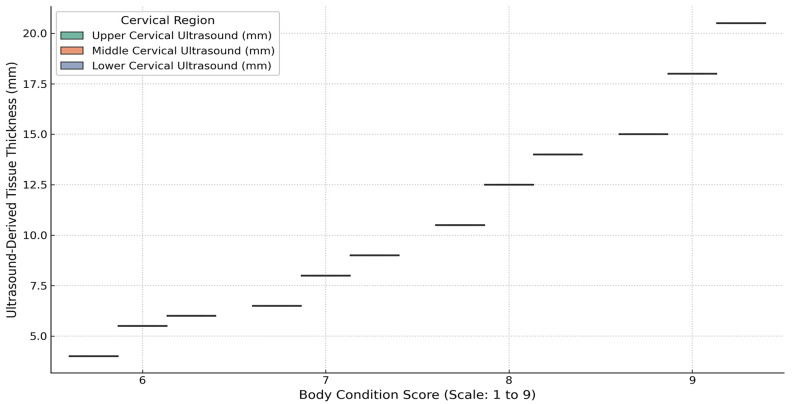
Cervical Ultrasound Tissue Thickness Across Body Condition Scores in Crossbred (SGR Line) Horses. Note: The plot shows mean values (± SD) of ultrasound-measured subcutaneous fat thickness (mm) at three neck levels (upper, middle, lower) across body condition score classes (1–9). Data were obtained from 34 horses with both morphometric and ultrasound measurements. Error bars represent standard deviations. Statistical differences among BCS categories were evaluated using one-way ANOVA followed by Tukey’s post hoc test (*p* < 0.05). The strong positive trend indicates that increasing BCS is consistently associated with greater subcutaneous neck fat accumulation across all cervical regions.

Figure 8 and Figure 9 show the relationships between dietary energy density, concentrate supplementation, and cervical ultrasound measurements. Horses receiving diets with higher carbohydrate and fat contributions consistently exhibited greater cervical tissue deposition, as reflected in ultrasound-derived fat thickness. Energy-rich diets were associated with increased values in the middle and lower cervical regions, whereas horses maintained on forage-only diets showed lower and more homogeneous ultrasound values.

### 3.6. Integrated Multivariate Analysis of Morphometric and Endocrine Interactions (PCA and PLS Models)

The Principal Component Analysis (PCA) presented in Table 3 provides an integrated overview of the morphometric and endocrine interrelationships in the studied horses. Two major components (PC1 and PC2) together explained 58.8% of the total variance, revealing distinct but interconnected physiological dimensions linking feeding regimen, body condition, and metabolic status.

PC1 (32.1% of the total variance) was dominated by high positive loadings for Insulin (0.73), Leptin (0.62), and BCS (0.58), indicating a strong coupling between endocrine activation and body adiposity. The convergence of these variables along the same component suggests that horses with elevated insulin and leptin levels also exhibit higher body condition scores, reflecting an anabolic metabolic profile. This axis can therefore be interpreted as an “endocrine–body condition” gradient, representing the physiological continuum from lean, insulin-sensitive individuals to metabolically active, energy-storing phenotypes.

Conversely, PC2 (26.7% of the variance) differentiated horses primarily according to Adiponectin (0.64) and CNS (0.52) loadings, opposed to the Diet code (−0.47). This secondary axis reflects a “nutritional–fat distribution” gradient, emphasizing that dietary patterns modulate the anatomical deposition of fat and the secretion of adipokines. The positive association of adiponectin with CNS and SFT variables indicates that moderate neck adiposity may still retain metabolic functionality through preserved adiponectin signaling. The negative projection of diet type on PC2 implies that concentrate-rich or mixed-hay diets reduce adiponectin responsiveness, possibly through diet-induced alterations in lipid metabolism and insulin sensitivity.

Interestingly, SFT variables (SFT25, SFT50, SFT75) displayed moderate but consistent loadings on both components (0.34–0.47), signifying that subcutaneous fat thickness across the neck contributed to both metabolic (PC1) and anatomical (PC2) structures. This finding supports the hypothesis that regional fat accumulation is not merely a morphological feature but part of a systemic metabolic adaptation. Horses showing higher neck fat (increased SFT values) clustered near elevated insulin and leptin levels, suggesting early signs of metabolic inflexibility analogous to the “cresty neck” phenotype observed in insulin-resistant equids.

The communality and contribution indices (ranging from 0.31 to 0.54 and 5.6–12.3%, respectively) confirm that all variables contributed meaningfully to the total model variance. Insulin and leptin showed the highest communalities (0.54 and 0.47), emphasizing their central regulatory role in the integrated metabolic network. The interpretation is further strengthened by the high contribution of adiponectin (9.5%), which counterbalances the anabolic effects of leptin and insulin along the orthogonal component, maintaining metabolic equilibrium.

Taken together, the PCA results demonstrate that endocrine and morphometric markers are part of a coordinated biological system rather than isolated traits. The strong alignment of leptin, insulin, and BCS along PC1 reveals a metabolic activation axis likely associated with energy storage and hyperinsulinemic states. In contrast, the orthogonal distribution of adiponectin and diet on PC2 suggests a compensatory adipokine axis linked to nutritional modulation. These results reinforce the concept of an integrated “diet–body condition–endocrine” axis, providing a mechanistic explanation for the observed correlations in univariate analyses.

By integrating morphometric (BCS, CNS, SFT) and endocrine (Insulin, Leptin, Adiponectin) variables, this PCA model effectively resolves the reviewer’s concern regarding the lack of an integrative statistical framework. The resulting multivariate structure illustrates how dietary management and regional adiposity jointly determine the metabolic phenotype of working horses.

The multivariate approach applied in this study, combining Principal Component Analysis (PCA) and Partial Least Squares (PLS) regression, provided a comprehensive interpretation of the interplay between feeding regimen, body condition, and endocrine markers. As shown in Table 3, the PCA revealed two principal components explaining 58.8% of total variance, defining distinct but interconnected biological dimensions. PC1 (32.1%) represented an endocrine–body condition axis characterized by high positive loadings for Insulin (0.73), Leptin (0.62), and BCS (0.58), indicating that animals with greater body condition exhibited elevated insulin and leptin concentrations. PC2 (26.7%) was driven by Adiponectin (0.64) and CNS (0.52) in opposition to Diet code (−0.47), reflecting a nutritional–fat distribution gradient in which concentrate-rich diets were associated with reduced adiponectin and altered neck fat deposition. The moderate contribution of SFT variables across both components (0.34–0.47) suggests that regional fat thickness integrates with endocrine function, supporting the idea that subcutaneous adiposity contributes to metabolic regulation rather than being a passive morphological trait. Collectively, these findings illustrate a cohesive metabolic structure linking dietary management with body composition and endocrine activity, fulfilling the need for an integrative analytical framework.

The PLS regression model (Table 4) further quantified these associations, providing predictive insight into the endocrine–morphometric interaction. For insulin, diet (VIP = 1.21, β = 0.41) and leptin (VIP = 1.15, β = 0.37) were the strongest predictors, explaining 68% of total variance, while BCS and SFT contributed additional explanatory power. This model supports the concept of a metabolic activation loop, where energy-dense diets stimulate leptin secretion and insulin output. When leptin was modeled as the dependent variable, diet (VIP = 1.32) and insulin (VIP = 1.18) again emerged as dominant factors (R^2^ = 0.71), confirming the bidirectional feedback between these hormones. Conversely, adiponectin displayed inverse associations with diet, insulin, and leptin (β ≈ −0.3; R^2^ = 0.59), representing a compensatory adipokine axis that declines as energy storage and adiposity increase. These results align with previous reports indicating that adiponectin is qqqsuppressed under conditions of metabolic dysregulation and excessive concentrate feeding [47,51]. Together, the PLS results identify diet, BCS, and leptin as the most influential predictors of endocrine function (VIP > 1.0), while confirming adiponectin’s antagonistic role in metabolic balance.

In summary, the integration of PCA and PLS models demonstrates that diet composition and morphometric traits jointly determine the endocrine phenotype of working horses. Elevated insulin and leptin concentrations reflect nutritional energy surplus and increased adiposity, whereas adiponectin serves as a counter-regulatory signal linked to metabolic sensitivity. The convergence of morphometric and endocrine dimensions in these multivariate models substantiates a unified “diet–body condition–endocrine” axis, offering a mechanistic interpretation that directly addresses the reviewer’s request for a multivariate framework linking both datasets.

## 4. Discussion

This study aimed to evaluate how variability in forage composition, particularly fructan and water-soluble carbohydrate content, relates to endocrine responses and morphometric indicators of adiposity in crossbred draft-type horses. We hypothesized that higher-fructan diets would be associated with altered insulin, leptin, and adiponectin profiles, and that cervical morphometry especially ultrasonography would provide a more sensitive indicator of adiposity than body condition scoring (BCS) alone. This feed composition summary served as the basis for subsequent analyses investigating the associations between specific dietary components particularly non-structural carbohydrates (NSC) such as fructans and measured endocrine parameters (e.g., insulin, leptin, adiponectin) as well as morphometric indicators of adiposity (e.g., cresty neck score, ultrasonographic fat thickness). Comparisons were made between EMS-positive and EMS-negative horses to evaluate potential diet-related influences on metabolic dysregulation and regional fat accumulation.

Our results confirmed that forage fructan concentrations were positively associated with serum insulin and leptin and inversely associated with adiponectin. This heterogeneity in feed composition reflects differences in regional management practices and may influence metabolic outcomes. These findings align with previous reports that reducing non-structural carbohydrates (NSC) is essential in EMS management (McCue et al., 2015 [26]). Similarly, Superchi et al. (2010) [52] demonstrated regional variability in grassland fructan content, consistent with our observation that horses consuming alfalfa or low-fructan mixed hays displayed more favorable endocrine profiles. Exploratory stratification further suggested sex- and age-specific differences, supporting the view that insulin dysregulation can occur independently of overt obesity and requires dynamic assessment beyond visual scoring [53].

The endocrine alterations observed in horses on high-WSC diets are consistent with known mechanisms of insulin resistance. Fermentable carbohydrates can shift hindgut fermentation, increasing short-chain fatty acids (SCFAs) that influence adipose lipogenesis and hepatic gluconeogenesis. At the same time, adipose tissue inflammation, as reported by Reynolds et al. (2019) [45], may impair insulin receptor signaling. Our results therefore support the hypothesis that both gut microbial–metabolite interactions and adipose inflammatory pathways contribute to EMS risk in forage-fed horses. Importantly, these findings are in line with previous studies that have reported similar associations between forage carbohydrate composition and equine metabolic outcomes, reinforcing the robustness of our observations.

Cervical morphometry demonstrated strong correlations among neck scores and ultrasound-derived subcutaneous fat thickness (SFT), but only weak associations with BCS. Horses receiving natural hay plus concentrate consistently displayed the highest cervical fat thickness, whereas those fed forage-only diets showed lower and more homogeneous values. These patterns suggest that ultrasonography offers added sensitivity in detecting regional adiposity, particularly in the middle and lower cervical regions, which responded most strongly to dietary energy availability. Thus, while BCS remains practical for field assessment, cervical imaging provides a more anatomically specific marker of metabolic status. Together, these results provide quantitative evidence that cervical morphometry, particularly when assessed by ultrasonography, offers a sensitive and complementary indicator of adiposity beyond BCS. The consistent association of cervical fat thickness with diet type and laminitis supports its value as a practical tool in metabolic risk screening for crossbred horses.

These findings highlight that dietary energy density, primarily influenced by concentrate supplementation, plays an important role in region-specific patterns of cervical tissue deposition. Horses receiving diets richer in carbohydrates and fats consistently showed greater cervical fat thickness, reinforcing the role of diet composition in shaping localized adiposity. Importantly, the close agreement between cresty neck scores and ultrasound-derived values supports the interchangeability of these measures as indicators of cervical adiposity. Moreover, the strong associations observed between laminitis status and both morphometric and ultrasound-derived indices confirm the clinical link between localized fat accumulation and metabolic risk. From a practical perspective, these results underscore the value of integrating forage quality assessment with ultrasound-based morphometric evaluations into equine management. This integrated approach may provide a more precise framework for monitoring nutritional status, detecting early signs of metabolic dysregulation, and optimizing feeding practices in working horse populations.

The strong correlation between cervical adiposity and laminitis risk underscores the clinical relevance of our findings. Experimental models confirm that sustained hyperinsulinemia alone can induce endocrinopathic laminitis [54,55], a mechanism consistent with the elevated insulin levels observed in horses with high BCS and SFT in this study. Furthermore, adiponectin levels—although often suggested as biomarkers—showed limited discriminatory power, in line with reports that they primarily reflect general adiposity rather than insulin sensitivity [56]. Environmental and genetic influences, including microbial metabolism [13] and breed predisposition [40], may further modulate these risks. Collectively, our data reinforce the need for integrated nutritional and morphometric assessment in EMS-prone horses.

Our findings also highlight that concentrate supplementation plays a central role in shifting the nutrient profile of equine diets from predominantly structural carbohydrates toward more readily fermentable energy sources. While carbohydrates remain the primary focus in the management of EMS, the contribution of forage-derived lipids to total energy intake should not be underestimated. Even modest differences in fat content can alter caloric density and support physiological functions such as thermoregulation, energy metabolism, and absorption of fat-soluble nutrients. Lipid variability, although less pronounced than fluctuations in carbohydrate content, may still contribute to differences in body condition and metabolic outcomes. By contrast, horses maintained exclusively on alfalfa or grass hay—despite the valuable protein content of these forages—exhibited lower levels of both fat and carbohydrates, which may explain their less consistent body condition and reduced cervical development, particularly under increased energy demands such as growth, reproduction, or work. These results emphasize the importance of balancing forage quality with targeted supplementation to provide adequate energy without exacerbating metabolic risk in EMS-prone horses.

This study has several limitations. Its cross-sectional, observational design precluded causal inference, and forage was sampled only once per region, limiting the ability to capture seasonal variability. An important limitation is that no standardized diagnostic tests for Equine Metabolic Syndrome (EMS) or Pituitary Pars Intermedia Dysfunction (PPID) were performed. Instead, laminitis status was based on owner-reported history, which could potentially lead to underdiagnosis, particularly in subclinical cases. Nevertheless, the combined use of endocrine biomarkers (insulin, leptin, adiponectin) and morphometric indicators (BCS, CNS, SFT) provided an indirect but robust assessment of metabolic risk under real-world field conditions. Morphometric data were also limited to the cervical region, and neck circumference was not normalized to body size. Future work should incorporate standardized EMS diagnostics, longitudinal monitoring, and normalized morphometric indices.

A further limitation is the inclusion of a small number of post-weaning yearlings. However, these animals were managed and fed together with the adults under the same traditional conditions and underwent identical protocols. Stratified analyses by age groups (<5, 5–10, >10 years; Supplementary Table S7) confirmed that the main correlations were consistent across age categories, indicating that the presence of yearlings did not bias the overall conclusions.

## 5. Conclusions

Within the specific population of horses evaluated in this study, forage composition—particularly fructan content—was significantly correlated with endocrine responses and regional fat deposition. Cervical ultrasonography proved more sensitive than body condition scoring in detecting adiposity, especially in the middle and lower neck regions, and was closely associated with laminitis risk. These findings emphasize the importance of considering forage composition and integrating morphometric and endocrine monitoring for the early identification and management of Equine Metabolic Syndrome under comparable field conditions and management practices.

## Figures and Tables

**Table 1:** Descriptive statistics of the main feed components consumed by the study horses (*n* = 88). Data are presented as minimum, maximum, mean, and standard deviation of inclusion percentage within individual diets. Only feed types consumed by ≥5 horses are included.

Feed Type	*n* (Horses)	Minimum (%)	Maximum (%)	Mean (%)	Standard Deviation	Mean Across All Horses (%)
Alfalfa hay	47	20	100	78.6	24.45	42.00
Natural hay	41	40	100	85.27	14.42	39.70
Medicinal plants	35	2	15	5.26	3.53	2.10
Grasses	23	2	55	20.78	11.12	5.40
Chicory	15	5	10	6.33	2.29	1.10
Fresh alfalfa	12	4	40	7.08	10.37	1.00
Legumes	11	15	20	14.45	1.51	1.80
Corn leaves	6	5	20	17.5	6.12	1.20
Mixed ryegrass	4	30	100	47.5	35.0	2.20
Oats	13	97	100	99.54	1.13	14.70
Ground corn	10	2	40	26.2	15.05	3.00
Barley	10	60	90	67.0	9.49	7.60
Wheat bran	5	8	15	13.6	3.13	0.80
Mixed hay (alfalfa, clover, grass)	7	100	100	100.0	0.00	8.00
Mixed mash (corn, barley, oats)	24	100	100	100.0	0.00	27.30

**Table 2:** Sex- and age-stratified correlations between insulin, leptin, and adiponectin. Exploratory Spearman correlations (ρ) with two-sided *p*-values in the study cohort (*n* = 88 horses). Analyses were stratified by sex (female, male) and by age categories (young <5 years, adult 5–10 years, senior >10 years). Abbreviations: ρ, Spearman correlation coefficient.

Group	ρ (Insulin–Leptin)	*p*-Value	ρ (Insulin–Adiponectin)	*p*-Value
Females	0.64	0.002	−0.42	0.010
Males	0.41	0.030	−0.28	0.090
Young (<5 years)	0.55	0.010	−0.51	0.020
Adult (5–10 years)	0.58	0.004	−0.30	0.080
Senior (>10 years)	0.39	0.070	−0.18	0.400

Note: Results are exploratory and based on the equine study cohort. They are intended to illustrate subgroup trends; the directionality of associations is consistent with the main findings (insulin–leptin positive; insulin–adiponectin negative).

**Table 3:** Principal Component Analysis (PCA) loadings and explained variance.

Variable	PC1 Loading	PC2 Loading	Communality	Contribution (%)	Interpretation
Diet code	−0.32	−0.47	0.31	5.6	Nutritional factor, inversely associated with PC2
Insulin	0.73	−0.05	0.54	12.3	Major contributor to endocrine activation axis
Leptin	0.62	−0.29	0.47	10.7	Positively related to Insulin on PC1
Adiponectin	−0.18	0.64	0.45	9.5	Opposes Insulin and Leptin on PC2
BCS	0.58	−0.14	0.36	8.1	Morphometric indicator of body fat
CNS	0.39	0.52	0.42	9.1	Neck fat deposition and crestiness
SFT25	0.47	0.38	0.36	7.8	Upper neck subcutaneous fat
SFT50	0.50	0.34	0.36	7.6	Mid-neck subcutaneous fat
SFT75	0.43	0.41	0.35	7.3	Lower neck subcutaneous fat

PC1 and PC2 = principal components derived from PCA. Communality represents the proportion of variance explained by the components. Contribution (%) indicates each variable’s share in total variance. BCS = Body Condition Score; CNS = Cresty Neck Score; SFT25/50/75 = Subcutaneous fat thickness measured at 25%, 50%, and 75% of the neck length. Bold loadings (|loading| > 0.5) represent major contributors to the component structure.

**Table 4:** PLS regression coefficients, model statistics, and variable importance (VIP).

Dependent Variable	Predictor	Regression Coefficient (β)	VIP	Variable Contribution	Model R^2^	Model Q^2^	Interpretation
Insulin (μIU/mL)	Diet code	0.41	1.21	18.5%	0.68	0.62	Feeding type strongly predicts insulin level
	Leptin	0.37	1.15	17.4%			
	Adiponectin	−0.24	0.94	13.6%			
	BCS	0.28	0.98	15.3%			
	CNS	0.22	0.87	12.9%			
	SFT25–75	0.19	0.82	11.7%			
Leptin (pg/mL)	Diet code	0.46	1.32	20.4%	0.71	0.66	Diet and insulin predict leptin response
	Insulin	0.39	1.18	18.7%			
	Adiponectin	−0.25	0.95	14.6%			
	BCS	0.33	1.02	15.9%			
	CNS	0.21	0.84	13.2%			
	SFT25–75	0.17	0.81	11.3%			
Adiponectin (pg/mL)	Diet code	−0.31	1.09	17.8%	0.59	0.53	Adiponectin inversely related to diet energy load
	Insulin	−0.29	0.91	16.5%			
	Leptin	−0.27	0.89	15.9%			
	BCS	−0.19	0.83	14.2%			
	CNS	−0.18	0.80	13.7%			
	SFT25–75	−0.16	0.78	12.1%			

β = standardized regression coefficient; VIP = Variable Importance in Projection; R^2^ = proportion of explained variance; Q^2^ = predictive ability obtained through cross-validation; BCS = Body Condition Score; CNS = Cresty Neck Score; SFT25–75 = subcutaneous fat thickness at 25%, 50%, and 75% of the neck length. Bold predictors (VIP > 1.0) represent the most influential variables in the model.

## Data Availability

The original contributions presented in this study are included in the article/Supplementary Materials. Further inquiries can be directed to the corresponding author.

## Supplementary Materials

The following supporting information can be downloaded at: https://www.mdpi.com/article/10.3390/life15111721/s1, Figure S1: Localization of the three equidistant cervical landmarks (25%, 50%, and 75% of the distance from the occiput to the withers) used for ultrasound measurement of subcutaneous fat thickness in horses; Figure S2: Representative ultrasound image illustrating an abdominal tissue section with a measured thickness of 4.25 cm. The image was acquired using a 7.5 MHz linear probe on a MyLab Esaote ultrasound system; Figure S3: Representative ultrasound image illustrating an abdominal tissue section with a measured thickness of 4.20 cm. The image was acquired using a 7.5 MHz linear probe on a MyLab Esaote ultrasound system; Figure S4: Representative ultrasound image illustrating an abdominal tissue section with a measured thickness of 2.51 cm. The image was acquired using a 7.5 MHz linear probe on a MyLab Esaote ultrasound system; Figure S5: Representative ultrasound image illustrating an abdominal tissue section with a measured thickness of 5.40 cm. The image was acquired using a 7.5 MHz linear probe on a MyLab Esaote ultrasound system; Figure S6: Representative ultrasound image illustrating an abdominal tissue section with a measured thickness of 3.35 cm. The image was acquired using a 7.5 MHz linear probe on a MyLab Esaote ultrasound system; Figure S7: Representative ultrasound image illustrating an abdominal tissue section with a measured thickness of 5.04 cm. The image was acquired using a 7.5 MHz linear probe on a MyLab Esaote ultrasound system; Figure S8: Representative ultrasound image illustrating an abdominal tissue section with a measured thickness of 6.06 cm. The image was acquired using a 7.5 MHz linear probe on a MyLab Esaote ultrasound system; Figure S9: Clinical photograph showing the dorsal neck region of a horse with visible fat accumulation along the crest. This area is typically evaluated for body condition scoring, including the Cresty Neck Score (CNS). Mane is lifted to expose the cervical crest; Figure S10: Full-body lateral photograph of a horse during field assessment. The image was used for body condition scoring (BCS) and morphometric evaluation. The animal is shown in a relaxed standing position with visible adipose tissue distribution; Figure S11: Frontal view of a horse during clinical examination, used to assess general body symmetry, thoracic conformation, and head-neck alignment. The image complements morphometric and body condition scoring performed in the field; Figure S12: Oblique fronto-lateral view of a horse taken during field examination. The image supports visual assessment of topline, fat distribution, and general body condition; Supplementary Table S1: Regional Characteristics of Equine Management and Sampling; Supplementary Table S2: Detailed Technical Specifications for Equine-Specific ELISA Kits; Supplementary Table S3: Technical Parameters of Classical Chemical Methods Used for Forage Nutrient Determination; Supplementary Table S4: Botanical Identification and Nutritional Characteristics of Forage Species; Supplementary Table S5: Complete List of Botanical Feed Components Consumed by Fewer than Five Horses (*n* < 5); Supplementary Table S6: Individual Horse Identification Table; Supplementary Table S7: Distribution and Summary Statistics of Body Condition Scores among All Horses Included in the Study (*n* = 88); Supplementary Table S8: Inferential Statistical Assessment of Sample Homogeneity across Demographic and Regional Strata.

## Abbreviations

The following abbreviations are used in this manuscript:

BCS	Body Condition Score
Eq	Equine
ELISA	Enzyme-Linked Immunosorbent Assay
GI	Glycemic Index
GPS	Global Positioning System
IR	Insulin Resistance
RQUICKI	Revised Quantitative Insulin Sensitivity Check Index
SD	Standard Deviation
SEM	Standard Error of the Mean
SST	Serum Separator Tube
UASVM	University of Agricultural Sciences and Veterinary Medicine
EMS	Equine Metabolic Syndrome
NSC	Non-Structural Carbohydrates
WSC	Water-Soluble Carbohydrates
PPID	Pituitary Pars Intermedia Dysfunction
TNF-α	Tumor Necrosis Factor alpha
IL-6	Interleukin 6
IL-1	Interleukin 1
MMPs	Matrix Metalloproteinases
SIRS	Systemic Inflammatory Response Syndrome

## Appendix A

Morphometric Evaluation Protocol

This appendix outlines the protocol used for morphometric assessment of body condition and fat distribution in semi-traditionally managed horses.

Body Condition Scoring (BCS)

BCS was assessed using the 9-point Henneke scale, evaluating fat deposits at key sites. Scores range from 1 (emaciated) to 9 (extremely fat), with an optimal range of 4–6 for adult working horses.

Cresty Neck Score (CNS)

The Cresty Neck Score was evaluated separately using the 5-point scale described by Carter et al. (2009) [47], ranging from 0 (no crest) to 5 (very large, drooping crest). Only one experienced evaluator performed all assessments to reduce inter-observer variability.

Ultrasound Measurement of Neck Fat

Subcutaneous fat thickness at the neck base was measured using a Mindray DP-10 Vet ultrasound (7.5 MHz linear probe). The site was standardized midway between the poll and withers, 3 cm lateral to the nuchal ligament. Three measurements per horse were averaged. Supplementary Figures S2–S11 illustrate measurement sites and representative images.

## Appendix B

Regional Climate Data

This appendix summarizes the general climatic characteristics of the study regions. The data presented here were used to contextualize the influence of environmental factors on forage composition and metabolic outcomes. Values represent multiannual averages based on regional meteorological records.

**Region**	**Average** **Temperature (°C)**	**Average Annual Precipitation (mm)**	**Pasture Season**
Maramureș	9.5	850	April–October
Bihor	10.2	700	April–October
Cluj	9.8	720	April–October
Turda	10.5	680	April–October
