# Supplementary material for: Forage Carbohydrate Profiles and Endocrine Morphometric Interactions in Traditionally Managed Horses from Romania
Source: Life (Basel). 2025 Nov 6;15(11):1721. doi: 10.3390/life15111721 (PMC12653405; doi:10.3390/life15111721)
Supplement: Supplementary file 1 [file life-15-01721-s001.zip › life-3960868-supplementary.pdf]

life

MDPI

Supplementary Table S1. Regional Characteristics of Equine Management and Sampling in Northern and Central Romania

Region	Selection Criteria	Type of Units	Feeding Practices	Altitude (m)	Climate	Horses	Number of Horse Samples	Other Observations
Maramureș	High equine population density, agroecological diversity	Private households, semi-intensive units	Traditional feeding, seasonal access to natural pastures	200–600	Temperate–continental	Locally raised horses, semi-free, used for work and natural grazing	15	Clinical evaluations and sampling performed in the field to minimize stress
Bihor	High equine population density, agroecological diversity	Private households, semi-intensive units	Traditional feeding, seasonal access to natural pastures	200–600	Temperate–continental	Horses raised for work or transport, under traditional systems	18	Forage samples collected directly from daily administered feed
Cluj	High equine population density, agroecological diversity	Private households, semi-intensive units	Traditional feeding, seasonal access to natural pastures	200–600	Temperate–continental	Mixed local breeds, used for household tasks and riding	20	Morphometric assessments and blood sampling conducted under partially controlled field conditions
Turda	High equine population density, agroecological diversity	Private households, semi-intensive units	Traditional feeding, seasonal access to natural pastures	200–600	Temperate–continental	Horses managed semi-intensively, with variable	35	GPS coordinates collected but not

 https://doi.org/10.3390/life15111721

		access to pasture and local forage	disclosed due to data privacy

Supplementary Table S2. Detailed Technical Specifications for Equine-Specific ELISA Kits

Biomarker	Kit Name / Manufacturer	Catalog Number	LOD	Working Range	Intra-assay CV (%)	Inter-assay CV (%)	Recovery (%)	Dilution Applied
Insulin	Equine Insulin ELISA Kit – Mercodia AB (Sweden)	10-1113-01	0.025 µg/L	0.025–0.7 µg/L	5.8	9.2	96.3	None
Leptin	Horse Leptin ELISA – MyBioSource Inc. (USA)	MBS2507393	0.1 ng/mL	0.2–20 ng/mL	6.4	10.1	92.7	1:2
Adiponectin	Equine Adiponectin ELISA – Cloud-Clone Corp. (USA)	SEA605Eq	0.12 ng/mL	0.3–25 ng/mL	7.1	11.6	95.0	1:5

Supplementary Figure S1. Representative 4-PL Standard Curves

- Panel A: Standard curve for insulin quantification (Mercodia, 10-1113-01).
- Panel B: Standard curve for leptin quantification (MBS2507393).
- Panel C: Standard curve for adiponectin quantification (SEA605Eq).

All curves were fitted using a 4-parameter logistic (4PL) model in Gen5 Software (BioTek Instruments) with $R^2 > 0.998$.

Additional Notes

- All samples were run in duplicate, and results were averaged.
- Samples with OD readings outside the standard range were either diluted (as noted) or reanalyzed when volume permitted.
- Duplicate wells differing by >15% were flagged and repeated.

Supplementary Table S3. Technical Parameters of Classical Chemical Methods Used for Forage Nutrient Determination

Parameter	Fructans (Spectrophotometric)	Fat (Soxhlet Extraction)	Carbohydrates (Fehling Titration)
Sample Amount (g)	0.5 g ground forage (dry)	5 g ground forage (dry)	2 g ground forage (dry)
Solvent / Extractant	Distilled water (10 mL) at 80°C	Petroleum ether (boiling range 40–60°C)	Distilled water (100 mL)
Extraction Time	1 h at 80°C with intermittent agitation	6–8 h continuous extraction (Soxhlet apparatus)	1 h at room temperature with constant stirring
Enzymatic Step	Fructanase (Megazyme K-FRUC) enzymatic hydrolysis (60 min at 37°C)	–	–
Color Development Reagent	Phenol–sulfuric acid (or equivalent)	–	Fehling's A & B + methylene blue indicator
Measurement Wavelength	510 nm	Gravimetric measurement post-evaporation	Manual titration endpoint detection (color change)
Calibration Standard	Chicory inulin standard (Sigma-Aldrich, F8052)	N/A	D-glucose monohydrate standard
Replicates per Sample	2 (mean used for analysis)	2	2
CV Acceptance Threshold	≤10%	≤10%	≤10%

Step 1: Extraction

- 0.5 g dried, ground forage sample mixed with 10 mL distilled water.

- Incubated at 80°C for 1 hour with vortexing every 10 minutes.

Step 2: Enzymatic Hydrolysis

- 0.5 mL extract + 0.5 mL fructanase (Megazyme K-FRUC).
- Incubated at 37°C for 60 minutes.

Step 3: Colorimetric Detection

- Addition of phenol-sulfuric acid reagent.
- Absorbance measured at 510 nm using a UV-VIS spectrophotometer (e.g., Jasco V-630).

Step 4: Calculation

- Calibration curve built using known inulin concentrations (0–50 mg/L).
- Fructan concentration calculated from absorbance using linear regression ($R^2 > 0.995$).

Additional Notes

- Blanks and reagent-only controls were included for each analytical batch to account for baseline absorbance and contamination.
- Soxhlet extraction was performed with certified petroleum ether, and glassware was pre-rinsed with ethanol and dried at 105°C before use.
- Fehling titration was conducted with pre-standardized reagents prepared fresh weekly, using methylene blue (0.05%) to determine the endpoint (disappearance of blue color).
- For all chemical methods, samples exceeding a CV >10% between replicates were reanalyzed or excluded from statistical analysis.

Supplementary Table S4. Botanical Identification and Nutritional Characteristics of Forage Species

Scientific Name	Common Name	Forage Group	Fructan Potential	Nutritional Notes
Dactylis glomerata	Orchardgrass	Grass	High	Good digestibility; high NSC under stress
Lolium perenne	Perennial ryegrass	Grass	High	High fructan content in spring
Festuca arundinacea	Tall fescue	Grass	Moderate	Resistant to drought; moderate fructan levels
Trifolium pratense	Red clover	Legume	Low	Protein-rich; low in non-structural carbs
Trifolium repens	White clover	Legume	Low	Improves soil nitrogen; low fructan
Medicago sativa	Alfalfa	Legume	Negligible	High protein and calcium; low NSC
Plantago lanceolata	Ribwort plantain	Herb	Negligible	Moderate palatability; medicinal properties
Achillea millefolium	Yarrow	Herb	Negligible	Rich in secondary compounds; low NSC

Supplementary Table S5. Complete list of botanical feed components consumed by fewer than five horses (n < 5). Data are presented as minimum, maximum, mean, and standard deviation of inclusion percentage within individual diets. These components were excluded from the main table due to limited statistical representation but are retained here for descriptive completeness.

Feed Type	n (horses)	Minimum (%)	Maximum (%)	Mean (%)	Standard Deviation
Mixed ryegrass	4	30	100	47.50	35.00
Wheat bran	5	8	15	13.60	3.13
White clover	2	15	15	15.00	0.00
Plantago spp.	2	2	2	2.00	0.00
Spontaneous flora	1	30	30	30.00	-
Setarias spp.	1	3	3	3.00	-
Cat grass	1	20	20	20.00	-
Misc. foreign matter	2	3	3	3.00	0.00
Legume-grass mix (100 %)	2	100	100	100.00	0.00
Other (Horse #22 only)	1	10	10	10.00	-

Note: These plant components were identified in very few animals and were therefore not included in the main statistical analysis. Their presence, however, reflects the botanical diversity of forage sources and may provide context for regional or individual dietary practices.

Supplementary Table S6. Individual Horse Identification Table

Horse ID	Farm/ Region	Breed	Sex	Age (years)	Horse nutrition	Laminitis
Horse #1	Maramureș	SGR Crossbreed	M	3	alfalfa hay	0
Horse #2	Maramureș	SGR Crossbreed	F	3	alfalfa hay	0
Horse #3	Maramureș	SGR Crossbreed	M	2	alfalfa hay	0
Horse #4	Maramureș	SGR Crossbreed	M	2	alfalfa hay	0
Horse #5	Maramureș	SGR Crossbreed	F	1	alfalfa hay	0
Horse #6	Maramureș	SGR Crossbreed	F	2	alfalfa hay	0
Horse #7	Maramureș	SGR Crossbreed	F	2	alfalfa hay	0
Horse #8	Maramureș	SGR Crossbreed	F	2	alfalfa hay	0
Horse #9	Maramureș	SGR Crossbreed	F	2	alfalfa hay	0
Horse #10	Maramureș	SGR Crossbreed	F	3	alfalfa hay	0
Horse #11	Maramureș	SGR Crossbreed	F	1	alfalfa hay	0
Horse #12	Maramureș	Semi-heavy Draft Horse	F	2	grass hay	0
Horse #13	Maramureș	Semi-heavy Draft Horse	F	3	grass hay	0
Horse #14	Maramureș	Semi-heavy Draft Horse	F	2	mixed natural hay	0
Horse #15	Maramureș	Semi-heavy Draft Horse	Pregnant F	2	mixed natural hay	0
Horse #16	Maramureș	Semi-heavy Draft Horse	M	3	grass hay	0
Horse #17	Maramureș	Semi-heavy Draft Horse	M	2	grass hay	0
Horse #18	Maramureș	Semi-heavy Draft Horse	F	11	natural hay + concentrate	0
Horse #19	Maramureș	Semi-heavy Draft Horse	F	11	natural hay + concentrate	0
Horse #20	Maramureș	Semi-heavy Draft Horse	Pregnant F	3	mixed natural hay	0
Horse #21	Maramureș	Semi-heavy Draft Horse	M	3	mixed natural hay	0

Horse #22	Maramureș	Semi-heavy Draft Horse	Pregnant F	4	grass hay	0
Horse #23	Maramureș	Semi-heavy Draft Horse	M	2	alfalfa hay	0
Horse #24	Maramureș	Semi-heavy Draft Horse	Pregnant F	2	alfalfa hay	0
Horse #25	Oradea	SGR Crossbreed	F	4	alfalfa hay	1
Horse #26	Oradea	SGR Crossbreed	F	11	alfalfa hay	1
Horse #27	Oradea	SGR Crossbreed	F	6	alfalfa hay	0
Horse #28	Oradea	SGR Crossbreed	F	3	alfalfa hay	0
Horse #29	Oradea	SGR Crossbreed	F	7	alfalfa hay	1
Horse #30	Oradea	SGR Crossbreed	Pregnant F	14	mixed natural hay	1
Horse #31	Oradea	SGR Crossbreed	F	7	mixed natural hay	1
Horse #32	Oradea	SGR Crossbreed	F	4.5	natural hay	0
Horse #33	Oradea	SGR Crossbreed	F	8	grass hay	0
Horse #34	Oradea	Semi-heavy Draft Horse	Pregnant F	5	mixed natural hay	1
Horse #35	Oradea	Semi-heavy Draft Horse	Pregnant F	4.5	mixed natural hay	1
Horse #36	Oradea	Semi-heavy Draft Horse	F	4.5	mixed natural hay	0
Horse #37	Oradea	Semi-heavy Draft Horse	M	2	mixed natural hay	0
Horse #38	Oradea	Semi-heavy Draft Horse	F	5	mixed natural hay	0
Horse #39	Oradea	Semi-heavy Draft Horse	M	10	mixed natural hay	3
Horse #40	Oradea	Semi-heavy Draft Horse	Castrated M	6	mixed natural hay	1
Horse #41	Oradea	Semi-heavy Draft Horse	Castrated M	10	mixed natural hay	1
Horse #42	Oradea	Semi-heavy Draft Horse	Castrated M	6	mixed natural hay	2
Horse #43	Oradea	Semi-heavy Draft Horse	M	9	mixed natural hay	1
Horse #44	Oradea	Semi-heavy Draft Horse	F	4	mixed natural hay	0
Horse #45	Oradea	Semi-heavy Draft Horse	M	13	mixed natural hay	0
Horse #46	Oradea	Semi-heavy Draft Horse	M	1	mixed natural hay	0
Horse #47	Oradea	Semi-heavy Draft Horse	F	8	mixed natural hay	0
Horse #48	Oradea	Semi-heavy Draft Horse	F	5	mixed natural hay	0
Horse #49	Oradea	Semi-heavy Draft Horse	F	6	mixed natural hay	0
Horse #50	Oradea	Semi-heavy Draft Horse	F	6	mixed natural hay	0
Horse #51	Oradea	Semi-heavy Draft Horse	M	6	mixed natural hay	0
Horse #52	Oradea	Semi-heavy Draft Horse	M	2	mixed natural hay	0
Horse #53	Oradea	Semi-heavy Draft Horse	M	2	mixed natural hay	0
Horse #54	Oradea	Semi-heavy Draft Horse	M	9	mixed natural hay	0
Horse #55	Oradea	Semi-heavy Draft Horse	F	13	mixed natural hay	0
Horse #56	Oradea	Semi-heavy Draft Horse	F	4	mixed natural hay	0
Horse #57	Oradea	Semi-heavy Draft Horse	F	7	mixed natural hay	0
Horse #58	Oradea	Semi-heavy Draft Horse	F	0.5	mixed natural hay	0
Horse #59	Cluj	SGR Crossbreed	F	6	natural hay + concentrate	0
Horse #60	Cluj	SGR Crossbreed	F	3	natural hay + concentrate	0

Horse #61	Cluj	SGR Crossbreed	F	6	natural hay + concentrate	0
Horse #62	Cluj	SGR Crossbreed	F	2	natural hay + concentrate	0
Horse #63	Cluj	SGR Crossbreed	F	3	natural hay + concentrate	0
Horse #64	Cluj	SGR Crossbreed	M	2	natural hay + concentrate	0
Horse #65	Cluj	Semi-heavy Draft Horse	F	3	grass hay	0
Horse #66	Cluj	Semi-heavy Draft Horse	F	3	grass hay	0
Horse #67	Cluj	Semi-heavy Draft Horse	F	9	grass hay	0
Horse #68	Cluj	Semi-heavy Draft Horse	M	5	grass hay	0
Horse #69	Cluj	Semi-heavy Draft Horse	F	4	grass hay	0
Horse #70	Cluj	Semi-heavy Draft Horse	F	0.5	grass hay	0
Horse #71	Cluj	Semi-heavy Draft Horse	F	8	natural hay + concentrate	1
Horse #72	Cluj	Semi-heavy Draft Horse	F	4	natural hay + concentrate	0
Horse #73	Cluj	Semi-heavy Draft Horse	F	8	natural hay + concentrate	0
Horse #74	Cluj	Semi-heavy Draft Horse	F	3	natural hay + concentrate	0
Horse #75	Cluj	Semi-heavy Draft Horse	F	8	natural hay + concentrate	0
Horse #76	Cluj	Semi-heavy Draft Horse	F	5	natural hay + concentrate	0
Horse #77	Cluj	Semi-heavy Draft Horse	F	3	natural hay + concentrate	0
Horse #78	Cluj	Semi-heavy Draft Horse	F	3	natural hay + concentrate	0
Horse #79	Cluj	Semi-heavy Draft Horse	F	1	natural hay + concentrate	0
Horse #80	Cluj	Semi-heavy Draft Horse	M	5	natural hay + concentrate	0
Horse #81	Cluj	Semi-heavy Draft Horse	F	7	natural hay + concentrate	0
Horse #82	Turda	Semi-heavy Draft Horse	F	0.5	natural hay + concentrate	0
Horse #83	Turda	Semi-heavy Draft Horse	F	0.5	natural hay + concentrate	0
Horse #84	Turda	Semi-heavy Draft Horse	F	10	natural hay + concentrate	0
Horse #85	Turda	Semi-heavy Draft Horse	F	2	natural hay + concentrate	1
Horse #86	Turda	Semi-heavy Draft Horse	F	9	natural hay + concentrate	0
Horse #87	Turda	Semi-heavy Draft Horse	F	15	natural hay + concentrate	0
Horse #88	Turda	Semi-heavy Draft Horse	F	12	grass hay	0

Note: Supplementary Table S6 summarizes individual metadata for all 88 horses enrolled in the study. Unique anonymized identifiers (Horse #1 to Horse #88) were used for traceability across datasets while preserving confidentiality. Breed categories include *SGR Crossbreed* (a structured local crossbreeding population) and *Semi-heavy Draft Horse* (a robust native type used in mixed-purpose traction systems). Nutritional information was compiled based on owner-reported feeding practices corroborated by on-site inspection of feedstuffs. The variable "Laminitis" represents historical or current laminitic status, with binary encoding (1 = positive clinical history or signs; 0 = no evidence of laminitis).

Supplementary Table S7. Distribution and summary statistics of Body Condition Scores among all horses included in the study (n = 88).

Statistic	Value
Sample size (N)	88
Mean ± Standard Deviation (SD)	6.84 ± 1.43
Median (Interquartile Range, IQR)	7.0 (6.0–8.0)
Range (Min–Max)	4–9
First quartile (Q1)	6.0
Third quartile (Q3)	8.0
Mode	6
Horses with BCS ≥ 7 (%)	56.8%
Skewness	-0.17
Kurtosis	-0.88

Note. The table reports descriptive statistics for the Body Condition Scores (BCS) of the entire study population. Values are expressed as mean ± standard deviation (SD), median with interquartile range (IQR), and range (minimum–maximum). Additional parameters such as quartiles (Q1, Q3), mode, and distribution metrics (skewness, kurtosis) are included to provide a more comprehensive overview of the data. The percentage of horses with BCS ≥ 7 is also indicated, reflecting the proportion of individuals classified as overweight or obese according to widely used equine body condition scoring guidelines.

Supplementary Table S8. Distribution and summary statistics of Body Condition Scores among all horses included in the study (n = 88).Inferential statistical assessment of sample homogeneity across demographic and regional strata

Dependent Variable	Statistical Procedure	Fixed Factor / Covariate	Test Statistic (df)	p-Value	Interpretation
Age (years)	One-way Analysis of Variance (ANOVA)	Region (n = 4)	F(3,84) = 1.27	0.289	No significant inter-regional differences in mean age distribution
Age (years)	Mann–Whitney U Test	Sex (M/F)	U = 142.5	0.412	No sex-associated disparity in age structure
Insulin (µIU/mL)	Kruskal–Wallis H Test	Region (n = 4)	H(3) = 2.18	0.536	Regional variation in basal insulin concentration non-significant

Leptin (ng/mL)	Mann–Whitney U Test	Sex (M/F)	U = 158.0	0.368	Absence of sex effect on circulating leptin levels
Adiponectin (μg/mL)	Kruskal–Wallis H Test	Breed (n = 3)	H(2) = 3.04	0.281	No statistically significant breed-specific divergence
Body Condition Score (BCS)	One-way ANOVA	Region (n = 4)	F(3,84) = 0.96	0.414	Uniformity of mean BCS across regional subsets
Cresty Neck Score (CNS)	Kruskal–Wallis H Test	Breed (n = 3)	H(2) = 2.71	0.438	Homogeneous cervical adiposity distribution among breeds
Insulin (μIU/mL)	Spearman's Rank Correlation (ϱ)	Age (years)	ϱ = 0.12	0.378	Weak, non-significant monotonic association with age
Leptin (ng/mL)	Spearman's Rank Correlation (ϱ)	Age (years)	ϱ = 0.09	0.457	Weak, non-significant monotonic association with age
Adiponectin (μg/mL)	Spearman's Rank Correlation (ϱ)	Age (years)	ϱ = −0.11	0.421	Weak, non-significant inverse relationship with age

Note: Data normality was verified using the Shapiro–Wilk test, and homogeneity of variances via Levene's test. Parametric procedures (one-way ANOVA) were applied where assumptions were met; otherwise, non-parametric analogues (Kruskal–Wallis or Mann–Whitney) were employed to ensure robustness under non-parametric assumptions. Correlations between continuous covariates (e.g., age) and metabolic indicators were quantified using Spearman's rank correlation coefficients (ϱ). Across all analytical layers, none of the tested demographic (sex, breed, age) or regional factors exhibited statistically significant effects (all $p > 0.05$). These outcomes confirm the statistical homogeneity and structural comparability of the study population, justifying the subsequent use of pairwise correlation analyses as an exploratory framework under field conditions. Accordingly, these covariates were not retained as fixed effects in further modelling, given their negligible contribution to variance partitioning at the sample scale.

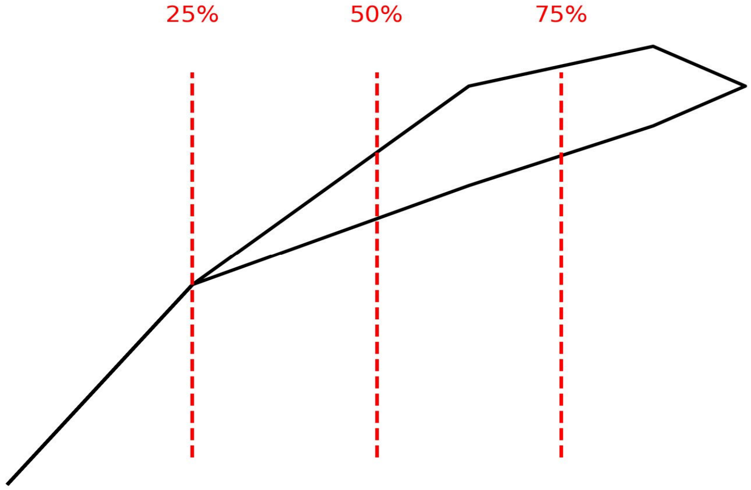

Figure S1. Localization of the three equidistant cervical landmarks (25%, 50%, and 75% of the distance from the occiput to the withers) used for ultrasound measurement of subcutaneous fat thickness in horses.

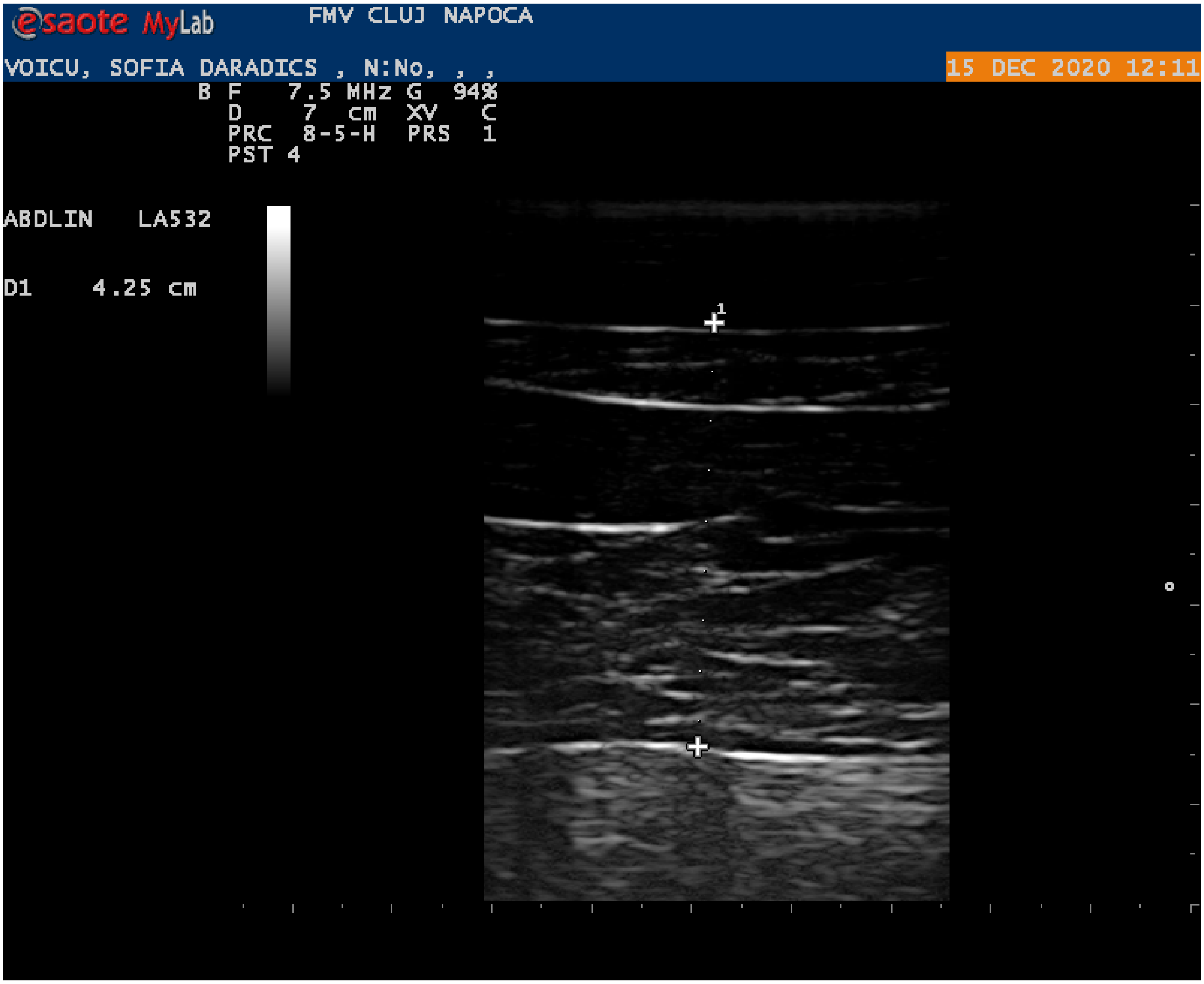

Figure S2. Representative ultrasound image illustrating an abdominal tissue section with a measured thickness of 4.25 cm. The image was acquired using a 7.5 MHz linear probe on a MyLab Esaote ultrasound system.

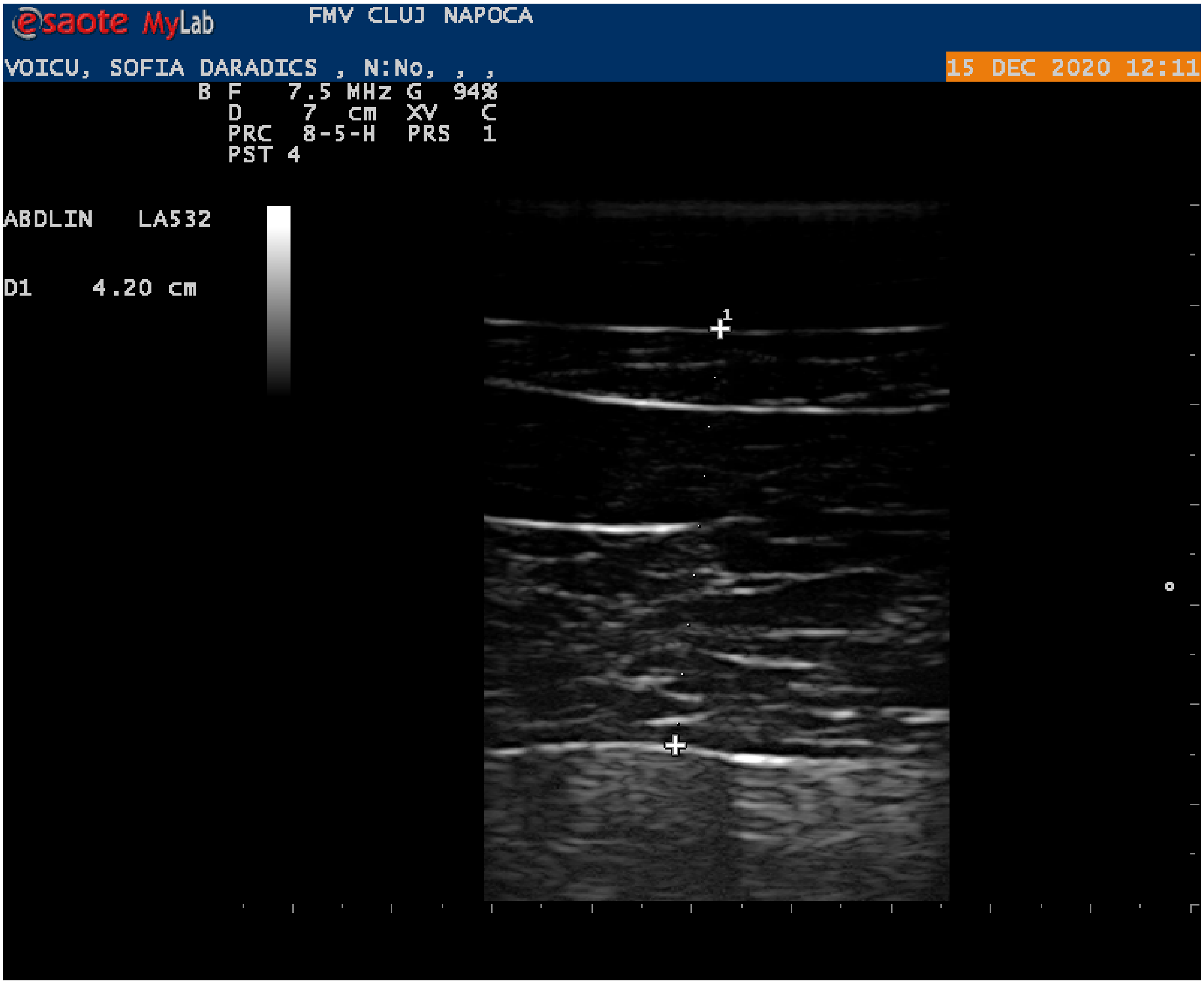

Figure S3. Representative ultrasound image illustrating an abdominal tissue section with a measured thickness of 4.20 cm. The image was acquired using a 7.5 MHz linear probe on a MyLab Esaote ultrasound system.

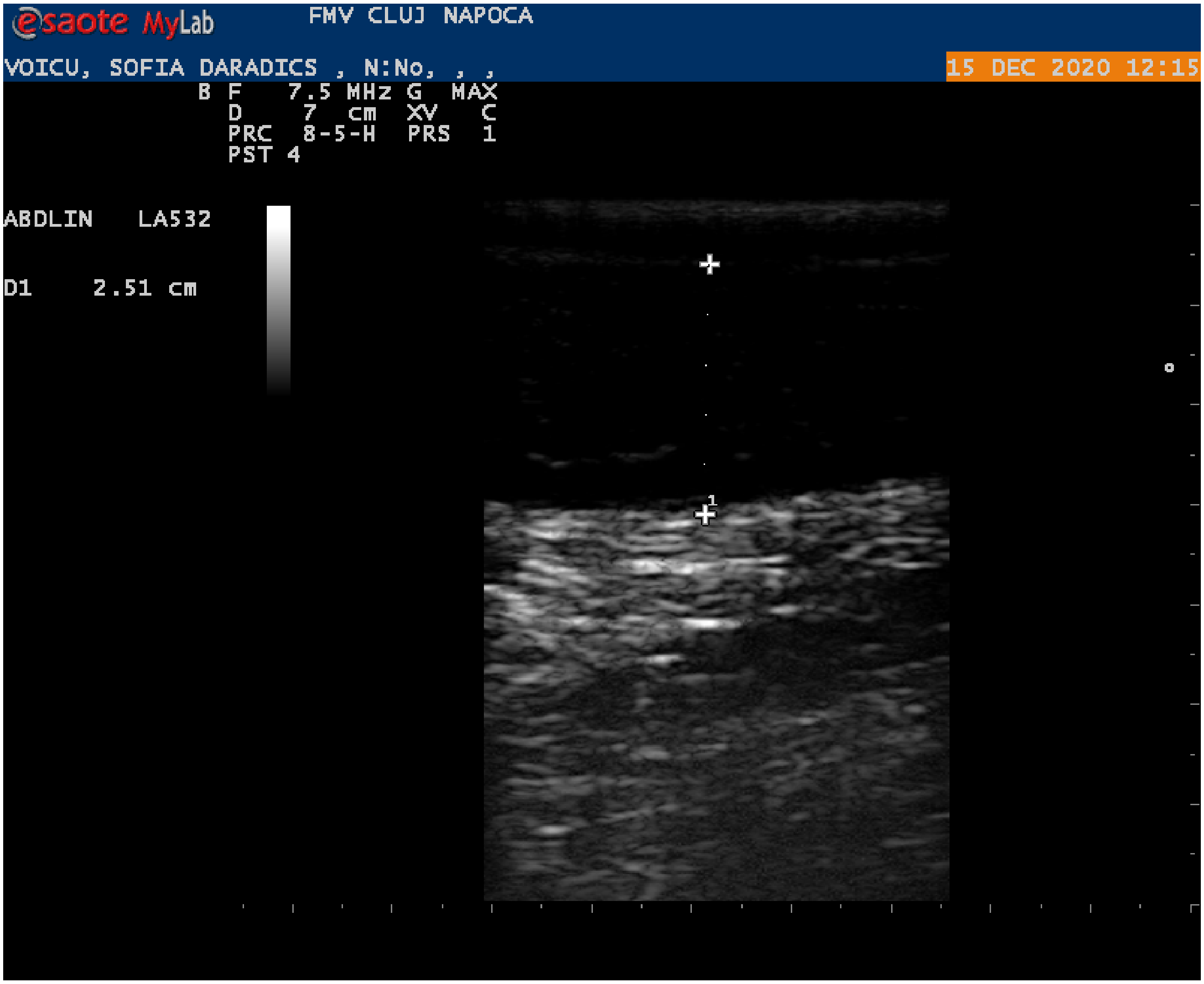

Figure S4. Representative ultrasound image illustrating an abdominal tissue section with a measured thickness of 2.51 cm. The image was acquired using a 7.5 MHz linear probe on a MyLab Esaote ultrasound system.

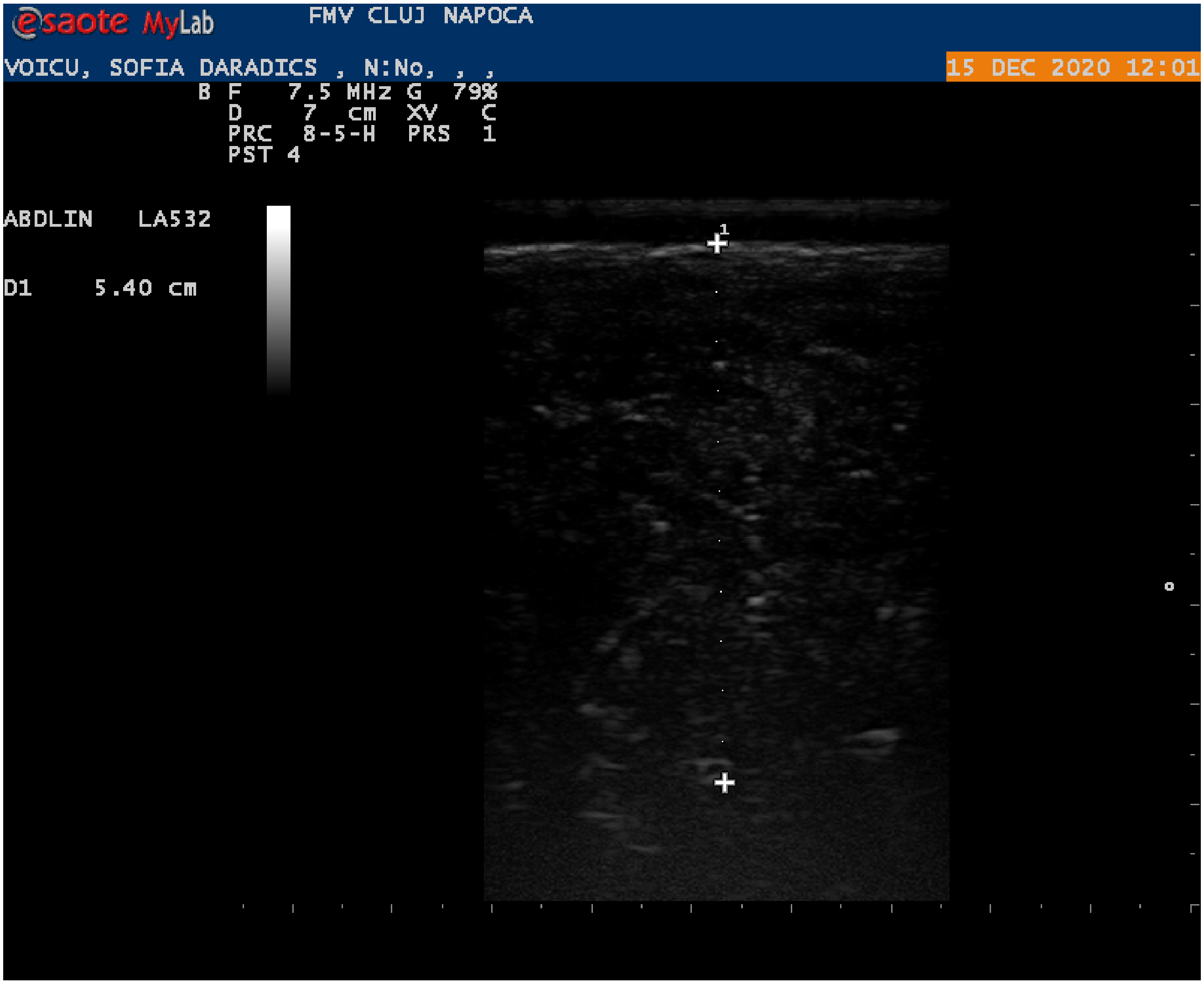

Figure S5. Representative ultrasound image illustrating an abdominal tissue section with a measured thickness of 5.40 cm. The image was acquired using a 7.5 MHz linear probe on a MyLab Esaote ultrasound system.

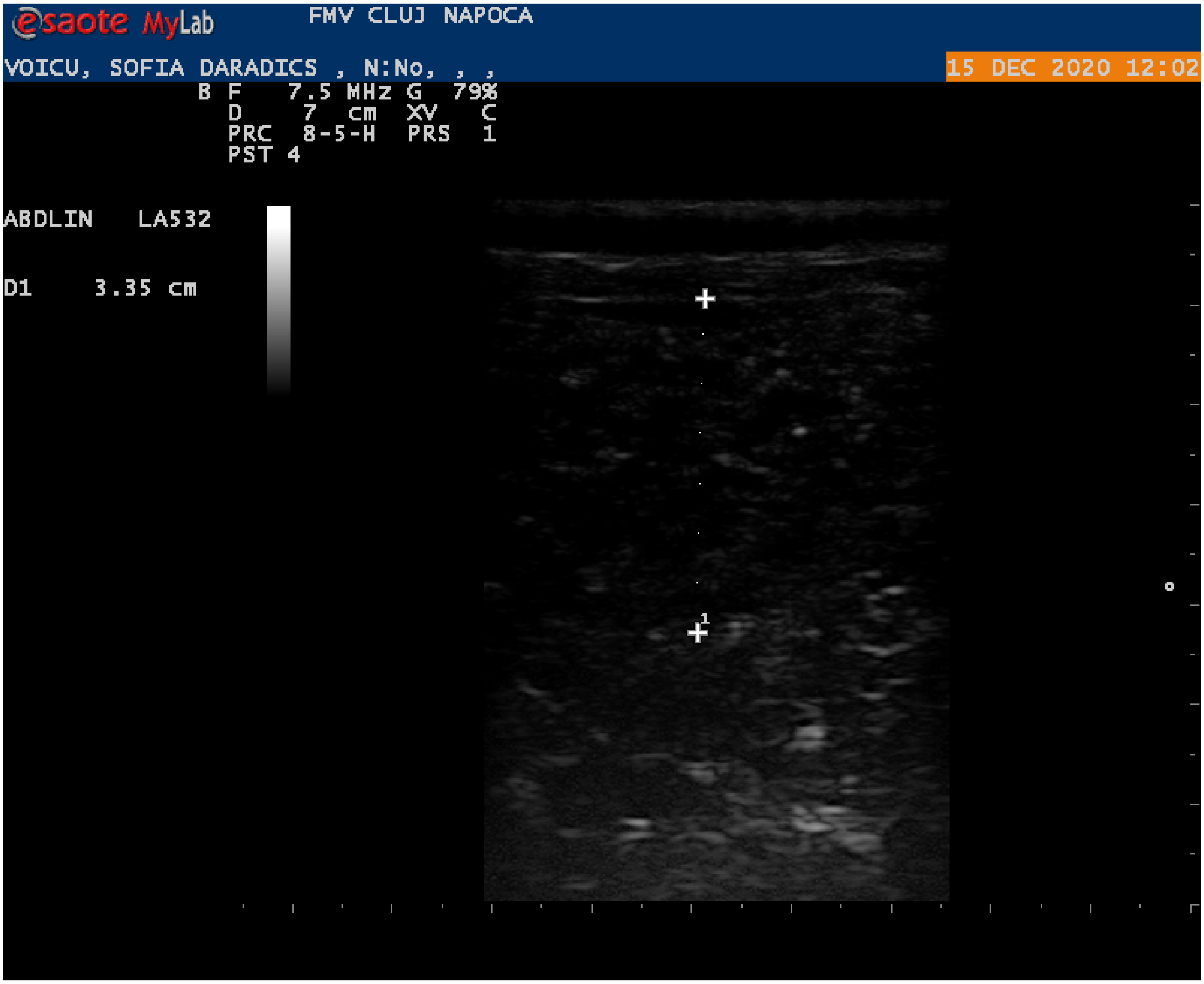

Figure S6. Representative ultrasound image illustrating an abdominal tissue section with a measured thickness of 3.35 cm. The image was acquired using a 7.5 MHz linear probe on a MyLab Esaote ultrasound system.

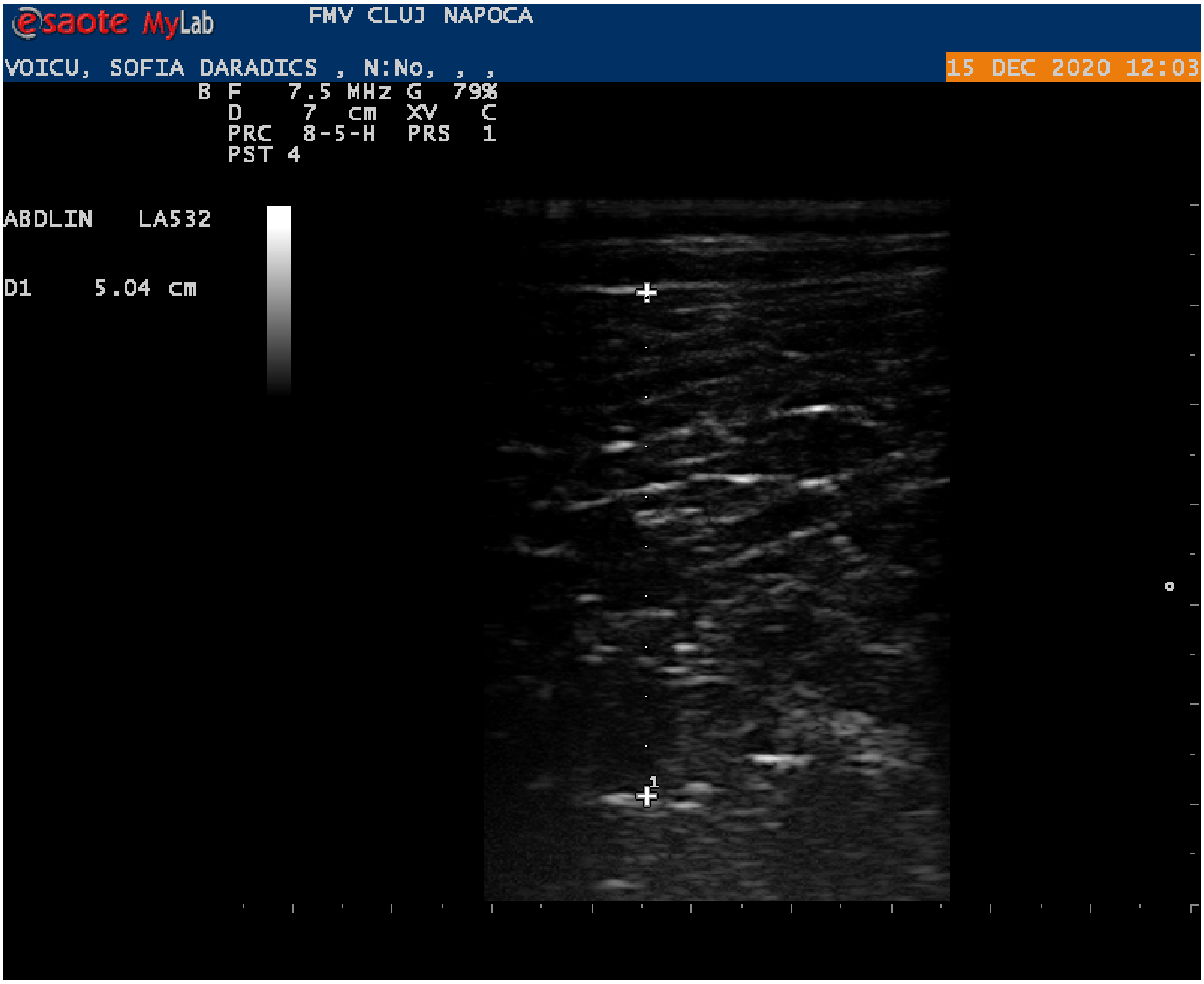

Figure S7. Representative ultrasound image illustrating an abdominal tissue section with a measured thickness of 5.04 cm. The image was acquired using a 7.5 MHz linear probe on a MyLab Esaote ultrasound system.

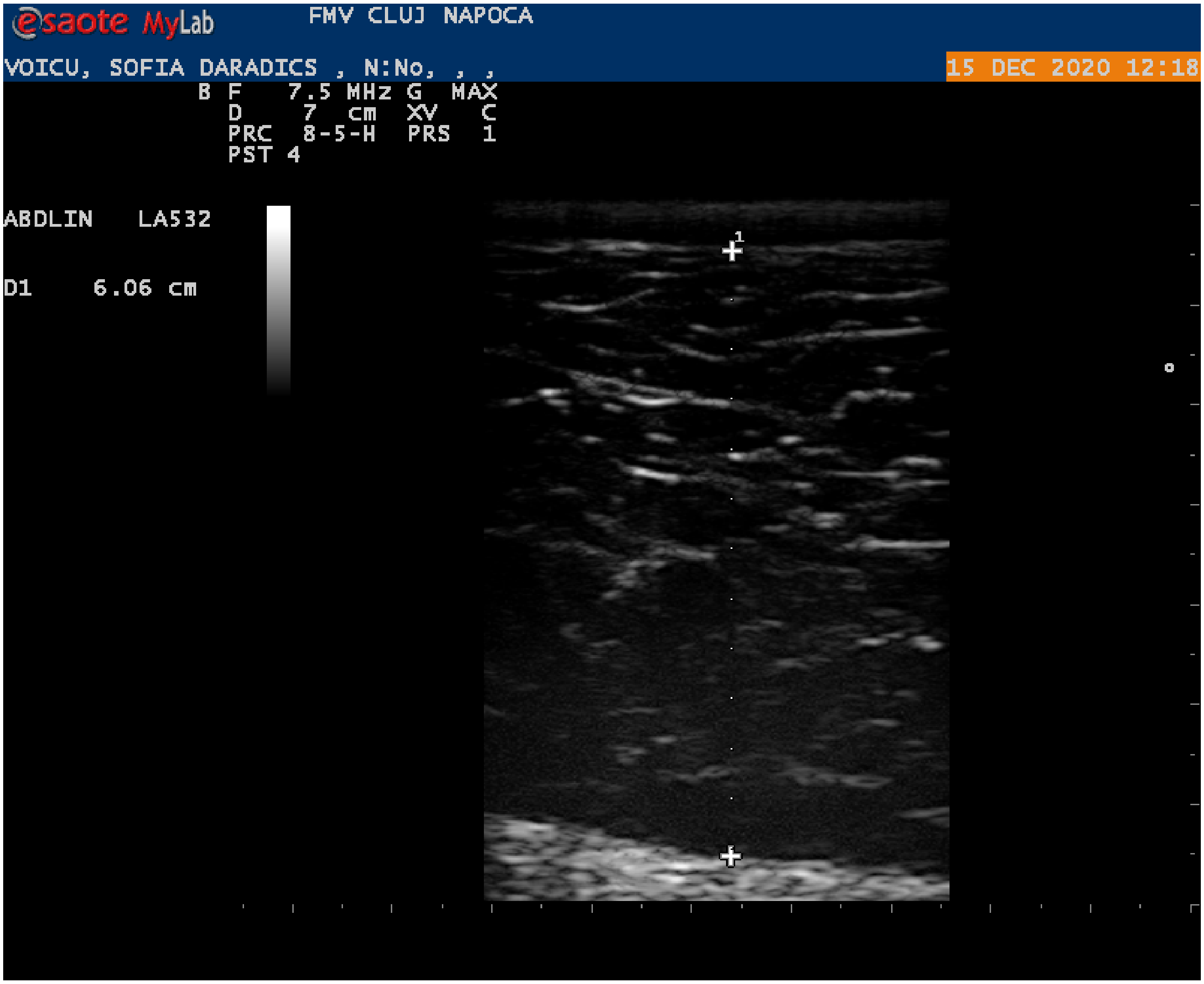

Figure S8. Representative ultrasound image illustrating an abdominal tissue section with a measured thickness of 6.06 cm. The image was acquired using a 7.5 MHz linear probe on a MyLab Esaote ultrasound system.

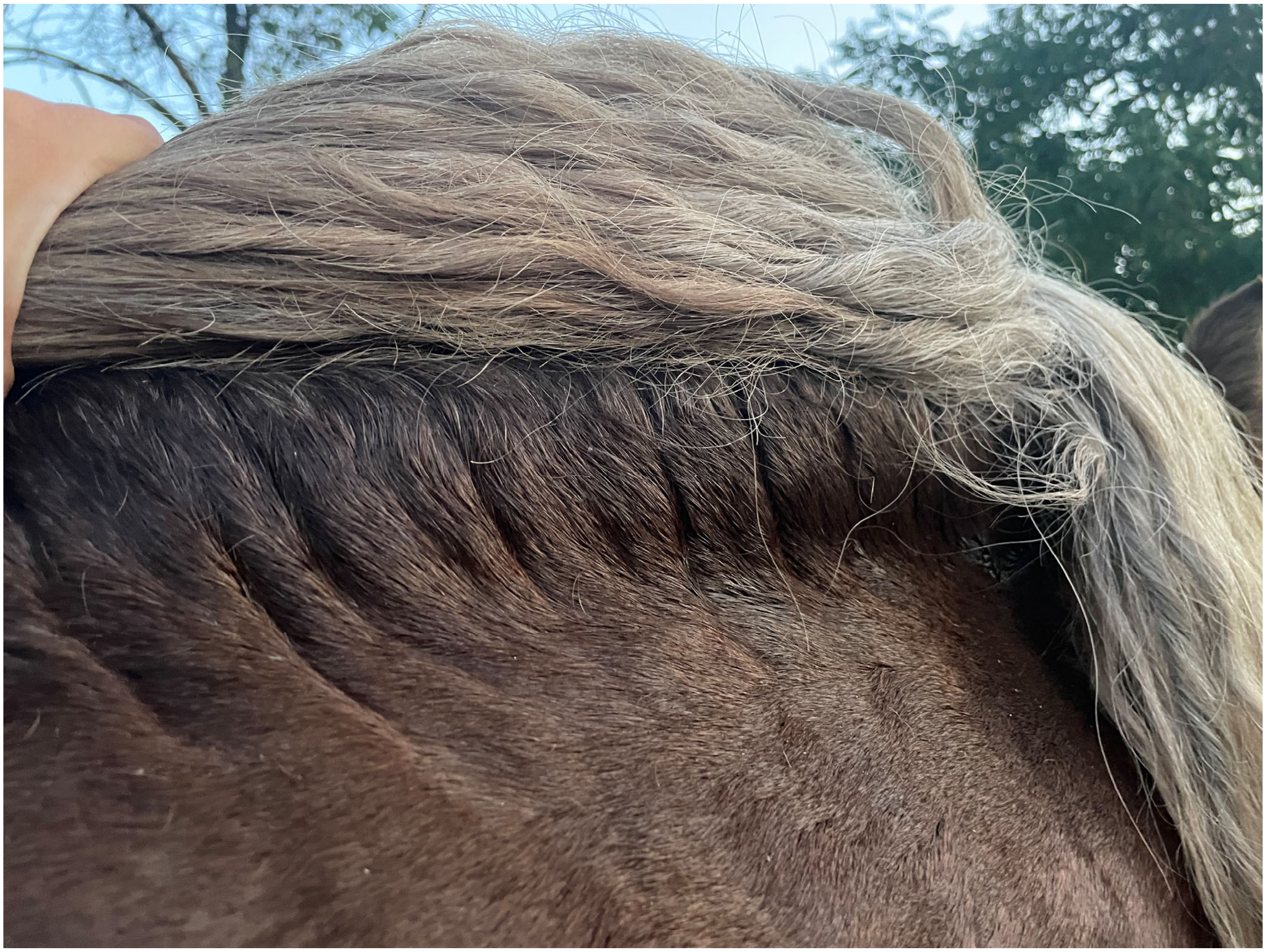

Figure S9. Clinical photograph showing the dorsal neck region of a horse with visible fat accumulation along the crest. This area is typically evaluated for body condition scoring, including the Cresty Neck Score (CNS). Mane is lifted to expose the cervical crest.

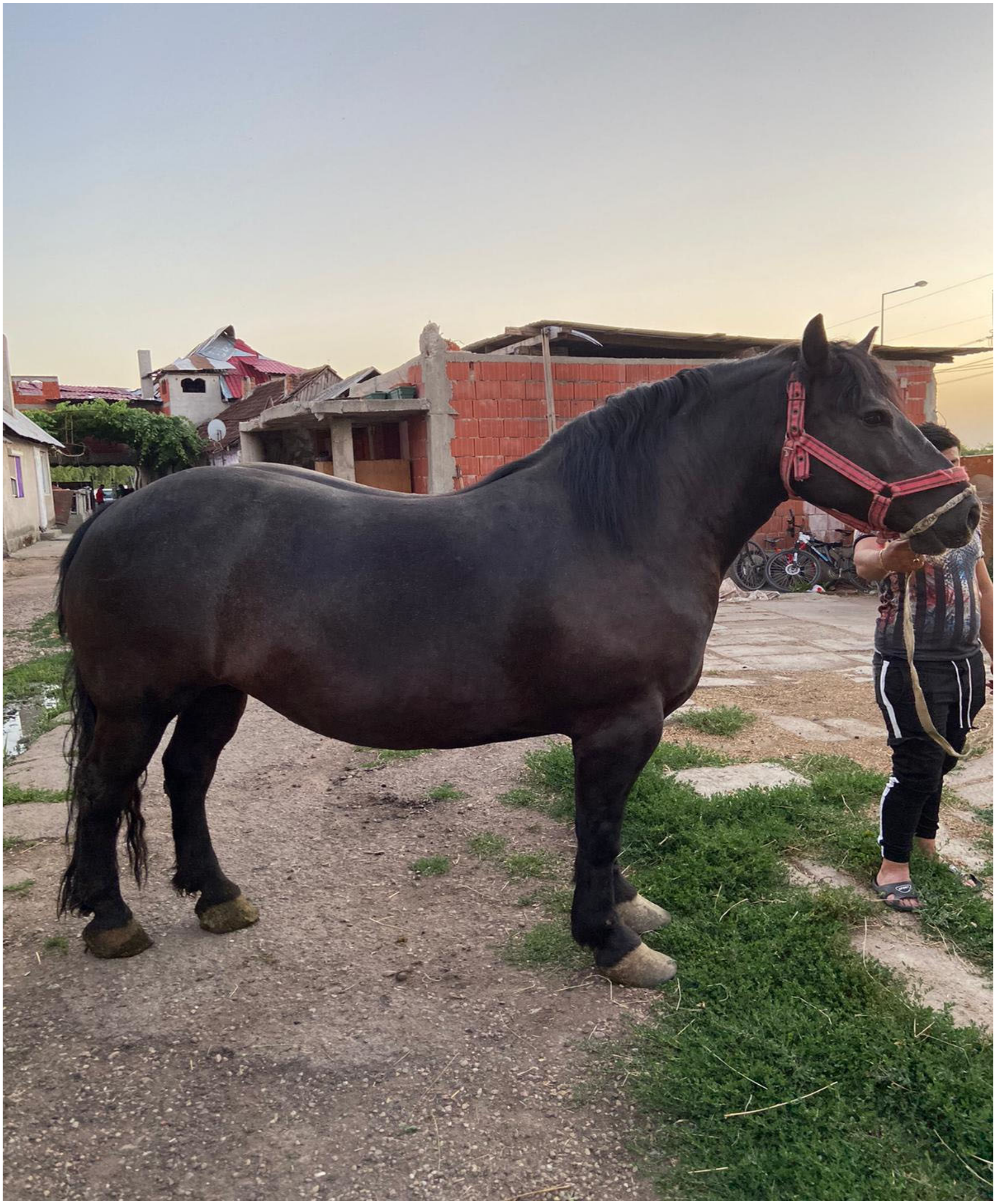

Figure S10. Full-body lateral photograph of a horse during field assessment. The image was used for body condition scoring (BCS) and morphometric evaluation. The animal is shown in a relaxed standing position with visible adipose tissue distribution across the neck, back, and abdomen.

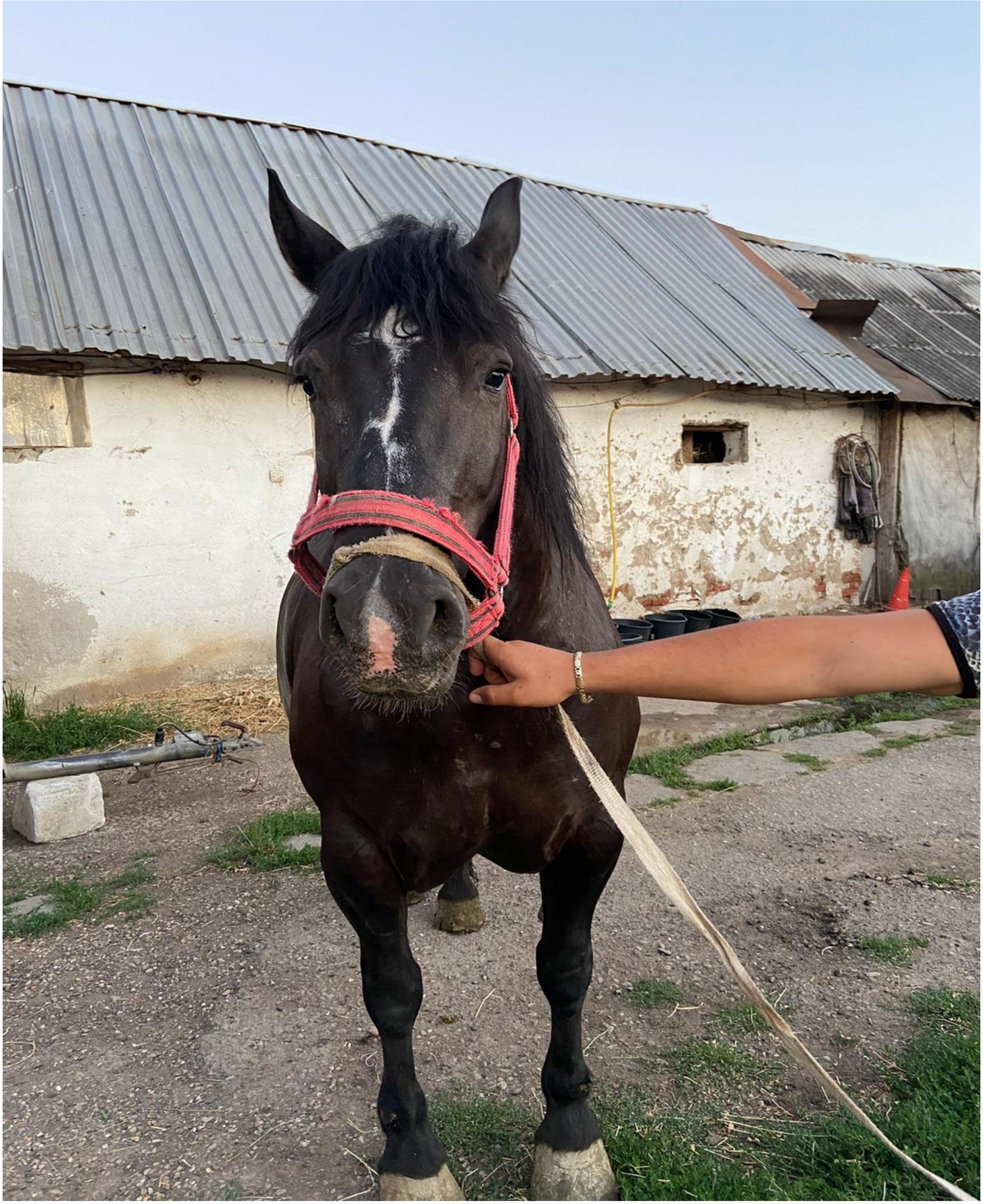

Figure S11. Frontal view of a horse during clinical examination, used to assess general body symmetry, thoracic conformation, and head–neck alignment. The image complements morphometric and body condition scoring performed in the field.

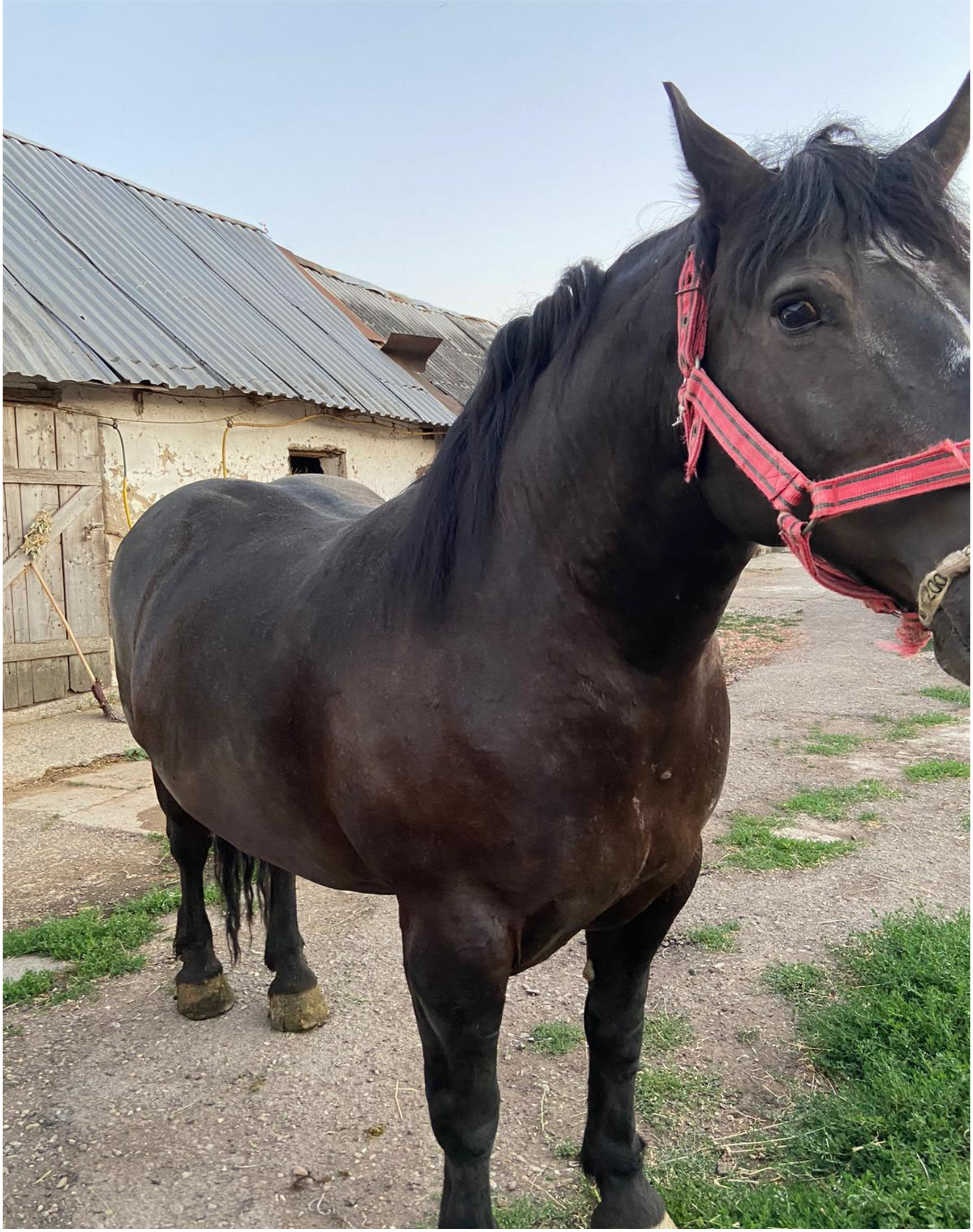

Figure S12. Oblique fronto-lateral view of a horse taken during field examination. The image supports visual assessment of topline, fat distribution, and general body condition.
